# Multiple Signals Converge on a Differentiation MAPK Pathway

**DOI:** 10.1371/journal.pgen.1000883

**Published:** 2010-03-19

**Authors:** Colin A. Chavel, Heather M. Dionne, Barbara Birkaya, Jyoti Joshi, Paul J. Cullen

**Affiliations:** Department of Biological Sciences, State University of New York at Buffalo, Buffalo, New York, United States of America; Stanford University School of Medicine, United States of America

## Abstract

An important emerging question in the area of signal transduction is how information from different pathways becomes integrated into a highly coordinated response. In budding yeast, multiple pathways regulate filamentous growth, a complex differentiation response that occurs under specific environmental conditions. To identify new aspects of filamentous growth regulation, we used a novel screening approach (called secretion profiling) that measures release of the extracellular domain of Msb2p, the signaling mucin which functions at the head of the filamentous growth (FG) MAPK pathway. Secretion profiling of complementary genomic collections showed that many of the pathways that regulate filamentous growth (*RAS, RIM101*, *OPI1,* and *RTG*) were also required for FG pathway activation. This regulation sensitized the FG pathway to multiple stimuli and synchronized it to the global signaling network. Several of the regulators were required for *MSB2* expression, which identifies the *MSB2* promoter as a target “hub” where multiple signals converge. Accessibility to the *MSB2* promoter was further regulated by the histone deacetylase (HDAC) Rpd3p(L), which positively regulated FG pathway activity and filamentous growth. Our findings provide the first glimpse of a global regulatory hierarchy among the pathways that control filamentous growth. Systems-level integration of signaling circuitry is likely to coordinate other regulatory networks that control complex behaviors.

## Author Summary

Signal integration is an essential feature of information flow through signal transduction pathways. The mechanisms by which signals from multiple pathways become integrated into a coordinated response remain unclear. We show that multiple pathways that regulate filamentous growth converge on a differentiation-dependent MAPK pathway. Our findings indicate that more extensive communication occurs between signaling pathways that control the filamentation response than has previously been appreciated. We suggest that global communication hierarchies regulate information flow in other systems, particularly higher eukaryotes where multiple pathways typically function simultaneously to modulate a complex response.

## Introduction

Signal transduction pathways regulate the response to extracellular stimuli. Complex behaviors frequently require the action of multiple pathways that act in concert to reprogram cell fate. In metazoan development for example, a highly regulated network of interactions between evolutionarily conserved pathways like Notch and EGFR coordinates every facet of cell growth and differentiation [1]. An important question therefore is to understand how different pathway activities are coordinated during complex behaviors. Addressing this question is increasingly problematic because signaling pathways operate in vast interconnected web-like information networks [2]. Miscommunication between pathways is an underlying cause of diseases such as cancer [3], and therefore it is both critically important and extremely challenging to precisely define the regulatory connections among signaling pathways.

The budding yeast *Saccharomyces cerevisiae* undergoes a variety of different responses to extracellular stimuli as a result of the function of evolutionarily conserved signal transduction pathways. In response to nutrient limitation, yeast undergoes filamentous growth [4],[5],[6], a cellular differentiation response in which changes in polarity, cell-cycle progression, and gene expression induce the formation of branched chains of interconnected and elongated filaments. The filamentous cell type is widely regarded as a model for differentiation [7],[8],[9],[10], and in pathogens like *Candida albicans*, filamentous growth is a critical aspect of virulence [11],[12],[13].

**Figure 1:**
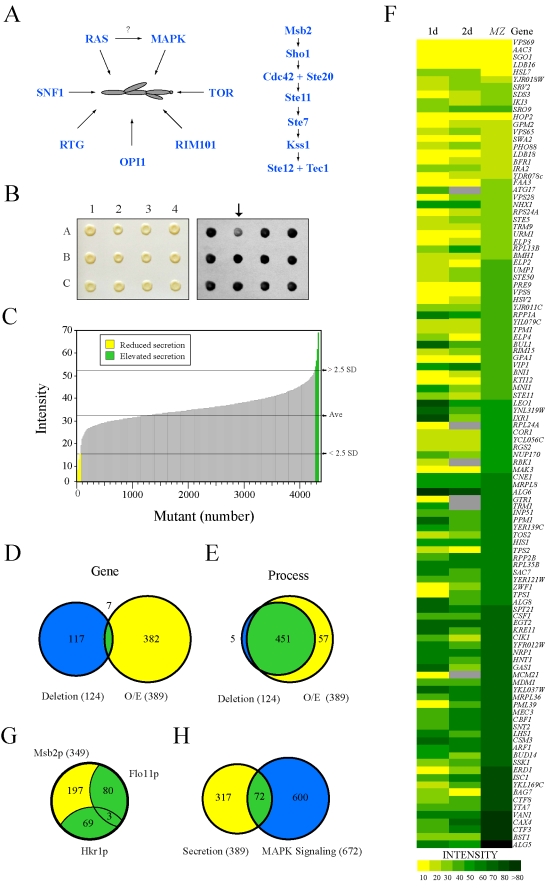
Secretion profiling of Msb2p. (A) Filamentous growth regulation in yeast. At left, different regulatory proteins and pathways that have been implicated in filamentous growth. It is unclear whether *RAS* regulates the FG pathway, as shown by the question mark. At right, the FG MAPK pathway. Cell surface proteins Msb2p and Sho1p connect to the Rho GTPase Cdc42p and effector p21 activated kinase Ste20p. The MAPKKK Ste11p, MAPKK Ste7p, and MAPK Kss1p regulate the activity of the transcription factors Ste12p and Tec1p. (B) Colonies from the *MAT* **a** deletion collection containing p*MSB2-HA* were pinned to nitrocellulose filters on SD-URA medium and incubated for 48h (left panel). Filters were rinsed in a stream of water and probed with anti-HA antibodies to detect shed Msb2p-HA (right panel). The mutant at position A2 (arrow) was identified as defective for Msb2p-HA secretion. (C) The secretion profile of Msb2p-HA in the haploid (*MAT* **a)** ordered deletion collection. Y-axis, normalized spot intensity (Intensity) as a measure of secreted Msb2p-HA. X-axis, deletion mutants ranked by normalized spot intensity. Yellow, mutants that showed a decrease (2.5 standard deviations below average, - 2.5 SD) in Msb2p-HA secretion. Green, mutants that showed elevated Msb2p-HA secretion (2.5 SD above average). (D) Overlap between genes identified in the deletion screen (blue circle at left, 124 genes) and the overexpression (O/E) screen (yellow circle at right, 389 genes). Seven common genes were identified (see Table S4C for details). (E) Overlap based on genes that share a common process/function. (F) Heat map showing normalized spot intensity (at 1d and 2d intervals) as a readout of Msb2p secretion compared to expression levels of an *MSB2-lacZ* (*MZ*) reporter. Yellow, reduced secretion; green, elevated secretion; grey, N/D. See Table S5 for details. (G) Comparative mucin secretion profiling. Genes that influence Msb2p-HA secretion when overexpressed (∼389 genes) were examined in strains containing Hkr1p-HA (PC2740) and Flo11p-HA (PC2043). Yellow, Msb2p-specific genes; blue, genes common to multiple mucins (Table S8). (H) Comparison between the secretion profile of Msb2p and regulators of a MAPK pathway growth reporter (*FUS1-HIS3*, [134]) whose expression is dependent upon the transcription factor Ste12p and elements of the *STE* pathway [135],[136],[137].

A number of different pathways are required for filamentous growth (Figure 1A, left panel). These include a MAPK pathway commonly referred to as the FG pathway (Figure 1A, right panel [14],[15],[16]), the *RAS* pathway [4],[17], the target of rapamycin or *TOR* pathway [18], the *RIM101* pathway [19],[20],[21], the retrograde pathway (*RTG* [7]), the inositol regulatory transcription factor Opi1p [22], and a global glucose control protein kinase Snf1p [23],[24],[25]. It is not clear whether these different pathways function together or independently to regulate filamentous growth. This question is compounded by the fact that several hundred other proteins have been implicated in the filamentation response [7],[26],[27].

To identify new aspects of filamentous growth regulation, we developed a screening approach to identify regulators of the MAPK pathway that controls filamentous growth. The FG pathway is regulated by the signaling mucin Msb2p [28], a cell-surface glycoprotein [29] that mediates signaling through the *RHO* guanine nucleotide triphosphatase (GTPase) Cdc42p [30]. Msb2p is processed in its extracellular domain by the aspartyl protease Yps1p, and release of the extracellular domain is required for FG pathway activation [31]. By measuring release of the extracellular domain of Msb2p in complementary genomic collections, we identified new regulators of the FG pathway. Unexpectedly, many of the major filamentation regulatory pathways (*RAS*, *RIM101*, *OPI1,* and *RTG*) were found to be required for MAPK activation. Our study indicates that the pathways that control filamentous growth are connected in co-regulatory circuits, which brings to light a systems-level coordination of this differentiation response.

## Results

### Secretion Profiling as an Approach to Identify FG Pathway Regulatory Proteins

To identify new regulators of the FG pathway, secretion of the extracellular domain of Msb2p [31] was examined using a high-throughput screening (HTS) approach in complementary genomic collections. Similar approaches have identified regulators of protein trafficking by mis-sorting and secretion of carboxypeptidase Y [32],[33],[34]. An ordered collection of 4,845 mutants deleted for nonessential open reading frames (ORFs, [35]) was transformed with a plasmid carrying a functional epitope-tagged *MSB2-HA* fusion gene, and transformants were screened by colony immunoblot to identify mutants with altered Msb2p-HA secretion (Figure 1B). Computational methods were used to quantitate, normalize, and compare secretion between mutants, which allowed ranking by the level of secreted Msb2p-HA (Table S3). As a result, 67 mutants were identified that showed reduced secretion of Msb2p-HA (Figure 1C, yellow), and 58 mutants were identified that showed elevated secretion (Figure 1C, green).

The secretion of Msb2p was also examined using an overexpression collection of 5,411 ORFs under the control of the inducible *GAL1* promoter [36]. This collection allows examination of essential genes and can be assessed in the Σ1278b background, in which filamentous growth occurs in an Msb2p- and MAPK pathway-dependent manner [37]. Approximately 390 genes were identified that influenced the secretion of Msb2p-HA when overexpressed (Table S4). The two screens identified few common genes (Figure 1D, 1.5% overlap), which is not entirely surprising given that gene overexpression does not necessarily induce the same (or opposite) phenotype as gene deletion [38] and because the two backgrounds exhibit different degrees of filamentous growth [37]. Significant overlap was observed at the level of gene process/function (Figure 1E, 89% overlap), which resulted in classification of genes into different functional categories (Figure S1; Tables S3 and S4). Introduction of an *MSB2-lacZ* reporter showed a high correlation between genes that affect Msb2p secretion and *MSB2* expression (Figure 1F; compare 1d to *MZ*). Because *MSB2* is itself a target of the FG pathway [28], many of the genes identified likely influence the activity of the FG pathway.

In total, 505 genes were identified that influenced Msb2p secretion, which might represent an underestimate due to the stringent statistical cutoff employed. This unexpectedly large collection suggests that Msb2p is subject to extensive regulation, although presumably many of these genes exert their effects indirectly. To enrich for genes that specifically regulate the FG pathway, secondary tests were performed. In one test, the secretion profile of Msb2p was compared to the secretion profile of two other mucins, the signaling mucin Hkr1p [39],[40] and transmembrane mucin Flo11p [41],[42]. Almost half the genes were common to multiple mucins (44%, Figure 1G) and may function in the general maturation of large secreted glycoproteins. In a second test, the secretion profile of Msb2p was compared to a genomic screen for genes that when overexpressed influence the expression of a FG pathway-dependent reporter (Figure 1H). These tests eliminated general regulators of mucin maturation/trafficking and enriched for potential MAPK regulatory proteins (∼72 candidate genes).

Several mutants were identified that were expected to influence Msb2p secretion. Mutants lacking FG pathway components (see Figure 1A), which are required for *MSB2* expression in a positive feedback loop [28], showed a defect in Msb2p-HA secretion (*ste20Δ, ste50Δ, ste11Δ*, and *ste7Δ*; Table S3). The mutant lacking the aspartyl protease Yps1p, which processes Msb2p and is required for release of the extracellular domain [31], was also identified (*yps1*Δ; Table S3). A subset of the genes that influence Msb2p secretion but not its expression might function through regulating expression of the *YPS1* gene, which is highly regulated [43]. The cell-cycle regulatory transcription factors Swi4p and Swi6p [44],[45],[46],[47],[48], were also found to regulate Msb2p secretion (Table S3B and S7). The *swi4* and *swi6* mutants had different phenotypes in Msb2p-HA secretion (Table S3), which suggests that the Swi4p and Swi6p proteins may play different roles in regulating cell-cycle dependent expression of the *MSB2* gene [49]. Some mating pathway-specific genes (*STE5*) were also identified, which may have an as yet unappreciated role in communication between the mating and FG pathways, which share a number of components [50].

As a proof-of-principle test, we disrupted fourteen genes that came out of the deletion screen in the Σ1278b background and tested for defects in Msb2p-HA secretion and FG pathway signaling. The test showed a >70% recovery rate based on phenotype and identified a novel connection between the tRNA modification complex Elongator and *MSB2* expression, establishing this protein complex as a novel regulator of the MAPK pathway [51]. Therefore, secretion profiling is a valid approach to identify established and potentially novel regulators of the FG pathway.

### Multiple Signaling Pathways Regulate the FG Pathway

**Figure 2:**
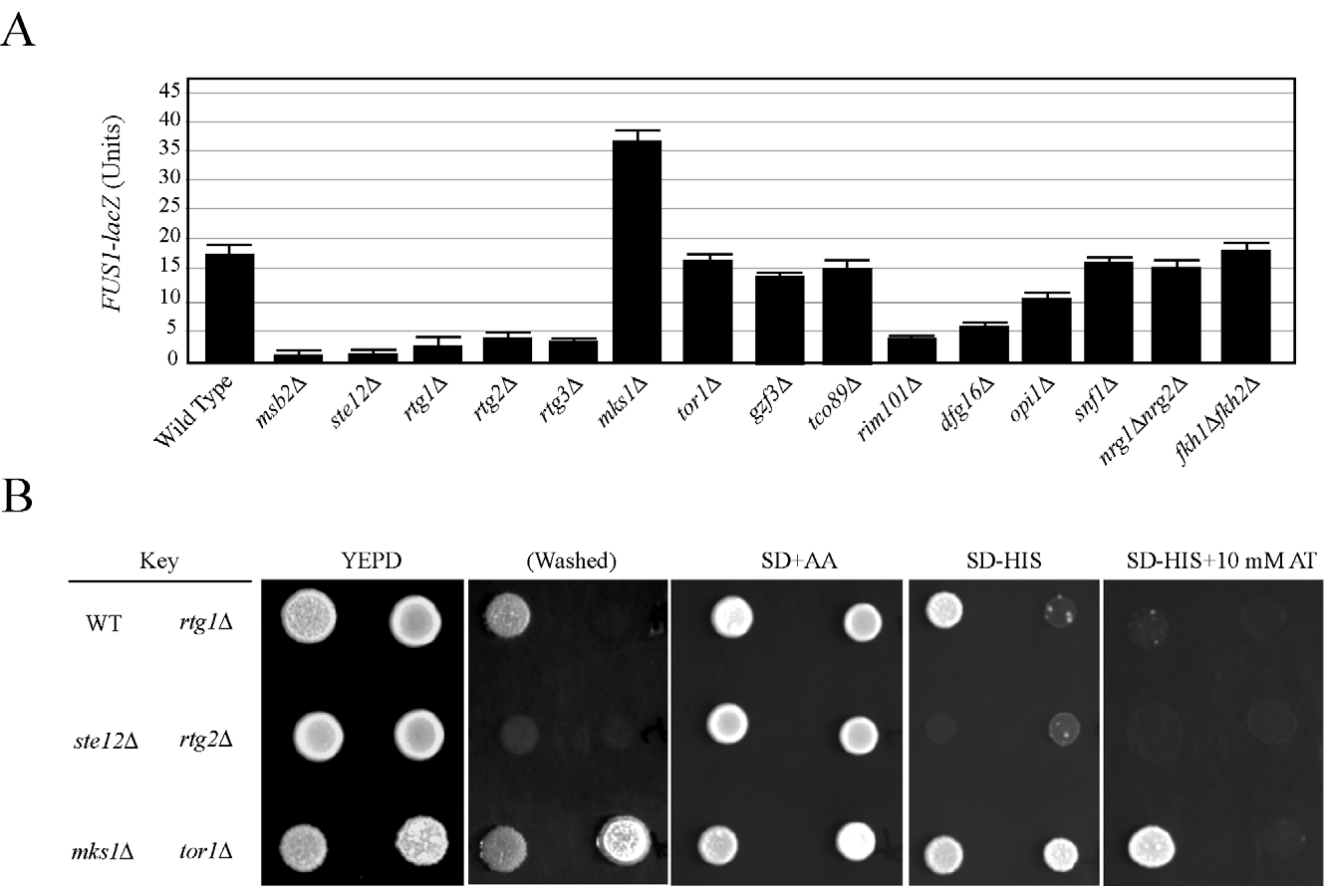
The role of filamentation control proteins on FG pathway activity. (A) *FUS1-lacZ* expression was monitored in the indicated strains in mid-log phase grown in YEPD medium. The experiment was performed in duplicate, and error bars represent standard deviation between experiments. In synthetic medium, the *opi1* mutant showed a >3-fold decrease in *FUS1-lacZ* expression compared to wild type (data not shown). (B) Comparison between *FUS1-HIS3* expression and invasive growth for a subset of mutants shown in (A). Equal concentrations of cells were spotted onto YEPD medium or synthetic medium containing glucose (SD) and all amino acids (AA), amino acids except histidine (-HIS), or amino acids except histidine and containing the competitive inhibitor 4-amino-1,2,4-triazole. After two days, the plates were photographed. The YEPD plate was photographed, washed in a stream of water, and photographed again (Washed). Growth on SD-HIS is indicative of MAPK pathway activity. Growth on SD-HIS+AT is indicative of hyperactivity. Although the *tor1* mutant induces hyperinvasive growth it does not influence MAPK activity.

To identify new genes that regulate the FG pathway, ∼50 candidate genes were disrupted in wild-type strains of the Σ1278b background, and the resulting mutants were tested for effects on FG pathway activity. To distinguish between mutants that influence filamentous growth from those that have a specific effect on FG pathway activity, a transcriptional reporter (*FUS1*) was used that in Σ1278b strains lacking an intact mating pathway (*ste4*) is dependent upon Msb2p and other FG pathway components including the transcription factor Ste12p (Figure 2A and 2B [28]). A number of potential MAPK regulatory proteins were identified by this approach, many of which have been implicated in regulating filamentous growth through their functions in other pathways.

Mitochondrial retrograde signaling (the *RTG* network), which is responsible for mitochondrial communication with the nucleus, was required for FG pathway signaling. The *RTG* network responds to the integrity of the mitochondria and nutrient state, specifically the availability of certain amino acids. The proteins Rtg1p, Rtg2p, and Rtg3p are the main targets of the pathway, and these factors initially induce the expression of genes involved in the TCA cycle [52]. Rtg1p, Rtg2p, and Rtg3p were required for FG pathway signaling (Figure 2A). These factors are under the control of Mks1p, which inhibits Rtg3p translocation to the nucleus, preventing expression of target genes [53]. Mks1p is subsequently under the control of Rtg2p, which will bind and sequester Mks1p and prevent its interaction with Rtg3p [54]. Consistent with its inhibitory role in the *RTG* pathway, Mks1p had an inhibitory role on FG pathway activity (Figure 2A and 2B). Rtg2p, in turn, is negatively regulated by the Tor1p complex via Lst8p [55]. Several mitochondrial components, including ribosomal subunits and enzymes of the TCA cycle, showed varied expression and signaling defects in the screen (Tables S3 and S4; Figure S7A). Deletion of Tor1p did not have the same effect as Mks1p (Figure 2A and 2B), but it has been shown that *RTG* can function independently of Tor1p inputs [56]. Consistent with a connection to *RTG*, the activity of the FG pathway was sensitive to certain amino acids such as glutamate (Figure S2).

Components of the Rim101p pathway [19], including the transcriptional repressor Rim101p and Dfg16p, which is required for processing and activation of Rim101p [20],[21], were required for FG pathway activity (Figure 2A). The Rim101p pathway is required for pH-dependent invasive growth, and the activity of the FG pathway was slightly sensitive to pH levels (Figure S2). Other regulators of the FG pathway included components of the *RAS* pathway (see below) and the inositol regulatory transcription factor Opi1p (Figure 2A [57],[58]), which has recently been tied to filamentous growth regulation [22].

The discovery that many filamentous growth regulatory pathways impinge on the FG pathway suggests a systems-level coordination between the pathways that regulate filamentous growth. We directly tested other filamentation regulatory proteins. Filamentous growth is regulated by the global glucose-regulatory protein Snfl1p [23],[24],[25]. Snf1p was not required for FG pathway activity (Figure 2A). The negative regulators Fkh1p/Fkh2p [59] and Nrg1p/Nrg2p [23],[60], which function to inhibit invasive growth, did not influence FG pathway activity (Figure 2A). Therefore, many but not all inputs into filamentous growth regulation also regulate the FG pathway. This finding may begin to account for the large number of genes identified by secretion profiling that impinge on the FG pathway.

### Ras2p/cAMP Regulates Starvation-Dependent Induction of *MSB2* Expression

**Figure 3:**
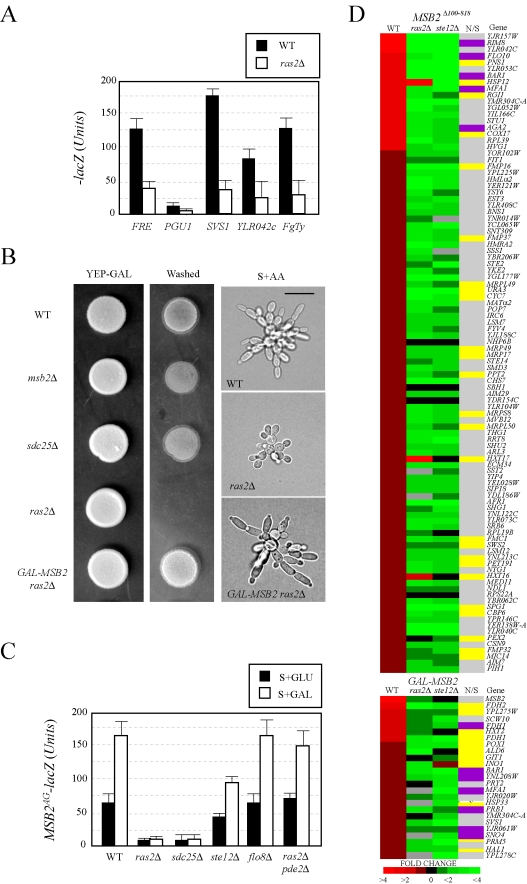
Components of the *RAS* pathway are required for *MSB2* expression and FG pathway regulation. (A) Expression of FG pathway-dependent reporters in wild-type cells and the *ras2Δ* mutant. Cells containing plasmid born *lacZ* fusions [65] were grown to mid-log phase in SD-LEU medium to maintain selection for the plasmids and harvested by centrifugation. β-galactosidase assays were performed in duplicate. Y-axis, β-galactosidase activity expressed in Miller Units (U). Error bars represent the standard deviation between experiments. (B) Invasive growth assays. For the plate-washing assay at left, equal concentrations of cells were spotted onto YEP-GAL medium and incubated for 2d. The plate was photographed (YEP-GAL) and washed in a stream of water to reveal invaded cells (Washed). For the single-cell invasive growth assay at right, cells were grown on S+GAL medium at low density for 16 hours and photographed at 100X. Bar, 10 microns. (C) *MSB2^AG^-lacZ* expression in wild type, *ras2Δ, sdc25Δ, flo8Δ, ste12Δ,* and *ras2Δ pde2Δ* mutants grown in SD or S+GAL medium. Y-axis, β-galactosidase activity expressed in Miller Units (U). The experiment was performed in duplicate, and error bars represent standard deviation between experiments. (D) Expression profiling of cells containing an activated FG pathway (either containing an activated allele *MSB2^Δ100–818^* or expressing *GAL-MSB2*) in wild-type cells the *ras2Δ* and *ste12Δ* mutants. Heat map of DNA microarray comparisons is shown. Red, fold induction; green, fold repression; black, no change; grey, N/D. Each spot represents the average of 3 independent comparisons. Genes involved in nutritional (N) scavenging or stress response (S) are marked in yellow (column N/S). Targets of the *RIM101* pathway are shown in purple [19]. The complete microarray dataset, including the raw data, statistical analysis, and functional classification of genes is presented in Table S6.

The *RAS* pathway has previously been implicated in FG pathway regulation [61]. However, systematic genomic analyses have failed to substantiate a connection between the two pathways [8],[9],[10],[62],[63],[64],[65], and a prevailing consensus is that the pathways function independently and converge on common targets [66],[67]. We found that the GTPase Ras2p [67],[68] was required for the expression of FG pathway-dependent reporters (Figure 3A), which indicates that the *RAS* pathway does regulate the FG pathway.

To determine where in the FG pathway that Ras2p functions, genetic suppression analysis was performed using alleles of *MSB2* and *SHO1* that hyperactivate the FG pathway [31]. Previous genetic suppression analysis indicates that Msb2p functions above Sho1p in the FG pathway [28] (Figure 1A). A hyperactive allele of *SHO1* (*SHO1^P120L^*) bypassed the signaling defect of *ras2Δ*, whereas the activated allele *MSB2^Δ100–818^* did not (Figure S3A and S3B), indicating that Ras2p functions above Sho1p and at or above the level of Msb2p in the FG pathway. This result led us to test whether Ras2p regulates the expression of the *MSB2* gene. Overexpression of *MSB2* (*GAL-MSB2*) bypassed the agar-invasion defect (Figure 3B, left panels) and the cell elongation defect of the *ras2Δ* mutant (Figure 3B, right panels; Figure S3A), which indicates that Ras2p regulates *MSB2* expression. We confirmed that Ras2p was required for the expression of an *MSB2*-lacZ reporter, including the starvation-dependent induction of *MSB2* expression (Figure 3C). This effect was independent of the FG pathway, which also regulates *MSB2* expression [28], because it was observed from a reporter lacking the Ste12p recognition sequence (Figure 3C, *MSB2^AG^-lacZ*) that makes *MSB2* expression Ste12p-insensitive (Figure 3C, *ste12*Δ). Secretion profiling also uncovered the alternative guanine nucleotide exchange factor Sdc25p [69],[70]. We confirmed that Sdc25p was required for starvation-dependent *MSB2* expression (Figure 3C).

Ras2p might regulate *MSB2* expression by modulating cAMP levels through activation of adenylate cyclase [71]. As shown in Figure 3C, bypass of the *MSB2* expression defect of the *ras2Δ* mutant was observed in cells lacking the phosphodiesterase *PDE2* [72]. The *ras2Δ pde2Δ* double mutant showed wild-type expression of the *MSB2* gene (Table S7), and overexpression of the *PDE2* gene inhibited FG pathway activity (Table S4). Sensitizing *MSB2* expression to cAMP levels represents a direct nutritional tie into MAPK regulation (Figure S2) and may explain why a large collection of genes that function in nutrient sensing were identified by secretion profiling (23.5%; Tables S3, S4, S5).

Ras2p also functions in a pathway that regulates the activity of the transcription factor Flo8p, which converges along with the FG pathway on the common target *FLO11* [8],[66],[73],[74],[75]. The Flo8p pathway is co-regulated by Ras2p and the glucose receptor-heterotrimeric G-protein Gpr1p-Gpa2p [76],[77],[78],[79],[80]. Gpr1p, Gpa2p, and Flo8p were not required for *MSB2* expression (Figure 3C, shown for *flo8*) or FG pathway signaling (Figure S2B). Ras2p also regulates a diverse collection of nutrient- and stress-related responses [81], and the *ras2Δ* mutant exhibits a number of phenotypes including glycogen accumulation, resistance to oxidative stress [82],[83], enhanced chronological lifespan [84],[85],[86], and resistance to oleate [87]. MAPK pathway mutants did not show these phenotypes (Figure S4A, S4B, S4C, S4D), which suggests that the FG pathway does not regulate Ras2p function. We therefore suggest that the two pathways function in a unidirectional regulatory circuit RAS -> MAPK.

Expression profiling using whole-genome DNA microarrays was used to validate the above results. The expression profiles of cells overexpressing *MSB2* or containing a hyperactive allele (*MSB2^Δ100–818^*) were compared in wild-type cells, the *ras2Δ* mutant, and the *ste12Δ* mutant genome wide (Figure 3D). Most of the genes that showed *MSB2-*dependent induction (Figure 3D, red) were reduced in the *ras2Δ* and *ste12Δ* mutants (Figure 3D, green). Expression profiling identified new targets of the FG pathway, including genes that function in nutrient scavenging, respiration, and the response to stress (Figure 3D; yellow, N/S, Nutrient/Stress). Targets of the *RIM101* pathway [19] were also identified (Figure 3D, purple). The induction of nutrient scavenging genes may represent a feed-forward loop where starvation induces FG pathway activation, which results in the induction of genes to sustain foraging. We note that the observed gene expression changes in the microarray profiling studies encompass both direct and indirect effects. To summarize, *RAS* regulates the FG pathway by regulating the starvation-dependent induction of *MSB2* expression.

### Rpd3p(L) Regulates *MSB2* Expression by Binding to the *MSB2* Promoter

**Figure 4:**
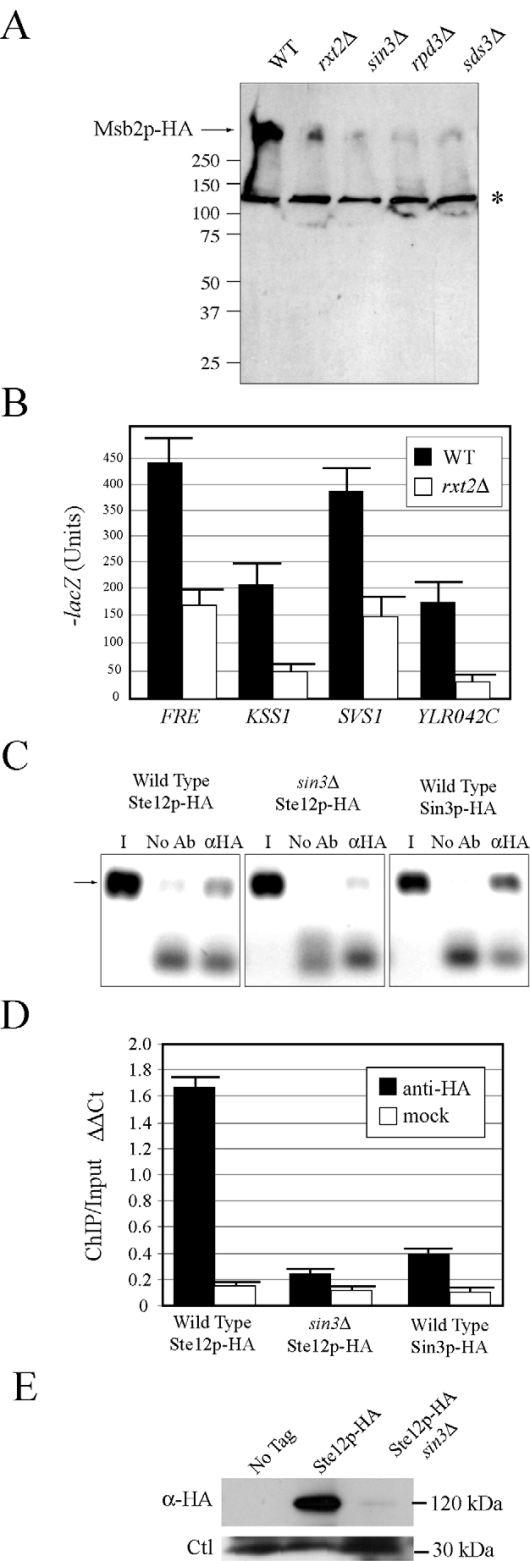
Rpd3p(L) promotes *MSB2* gene expression at the *MSB2* promoter. (A) Immunoblot of Msb2p-HA in wild-type cells and the *rxt2Δ, sin3Δ, rpd3Δ,* and *sds3Δ* mutants. The arrow refers to Msb2p-HA. The asterisk refers to a background band. (B) The activity of transcriptional (*lacZ*) reporters of the FG pathway in wild-type cells and the *rxt2Δ* mutant. β-galactosidase assays were performed in duplicate, and error bars represent standard deviation between replicates. (C) ChIP analysis of the *MSB2* promoter in wild-type cells or the *sin3Δ* mutant containing Ste12p-HA or Sin3p-HA fusion proteins. I, input; No Ab, no antibody control; αHA, anti-HA antibody. (D) Quantitation of the ChIP data. Graph shows relative levels of binding at the *MSB2* promoter relative to an intragenic region and to the *ACT1* gene as a control based on quantitative PCR analysis. ΔΔCt, threshold cycle; Bars correspond to the IP/Input ratio. (E) Immunoblot of Ste12p-HA in wild-type cells and the *sin3*Δ mutant. Ste12p-HA was immunoprecipitated to concentrate the protein. Ctl refers to a background band that served as a loading control.

To further explore the regulation of *MSB2* expression, transcriptional regulatory proteins identified by secretion profiling were examined. The Rpd3p(L) HDAC complex was identified as a strong positive regulator of *MSB2* expression. Rpd3p(L) comprises a 12 subunit complex [88] that includes the bridging proteins Sin3p [89],[90] and Sds3p [91]. Rpd3p, Sin3p, and Sds3p were required for starvation-dependent *MSB2* expression and to produce wild-type levels of the Msb2p protein (Figure 4A). Rpd3p, Sin3p, and Sds3p were required for the induction of FG pathway reporters (Table S7) and invasive growth (Figure S6A).

Rpd3p functions in distinct large and small complexes with different cellular functions. Rpd3p(S) recognizes methylated histone H3 subunits and functions to repress spurious transcription initiation from cryptic start sites in open reading frames [92]. Rpd3p(S) contains unique proteins Rco1p and Eaf3p. In contrast, Rpd3p(L) is enriched for different proteins including Rxt2p [92], which is required for Rpd3p(L) function [93]. Immunoblot analysis (Figure 4A), transcriptional reporters (Figure 4B), and invasive growth assays (Figure S6A), showed that Rxt2p was required for *MSB2* expression and FG pathway activation. In contrast, Rpd3p(L) was not required for pheromone response pathway activation (Figure S5), which shares components with the FG pathway but induces different target genes [5],[94],[95] and which does not require Msb2p function [28].

Histone deacetylases typically repress gene expression by causing compaction of chromatin into structures inaccessible to transcription factors. With respect to *MSB2* expression however, Rpd3p(L) had a positive role. We tested whether Rpd3p(L) negatively regulated an inhibitor of *MSB2* expression, such as the transcription factor Dig1p [96],[97],[98] but were unable to find evidence to support this possibility (Figure S6B). Rpd3p(L) functions as a positive regulator of some genes, including targets of the high osmolarity glycerol response (HOG) pathway by direct association with the promoters of HOG pathway targets [99], and in the regulation of some mating-specific targets [100]. Therefore, Rpd3p(L) might positively regulate *MSB2* expression by associating with the *MSB2* promoter. Chromatin immunoprecipitation (ChIP) analysis identified Rpd3p(L) at the *MSB2* promoter (Figure 4C, Sin3p-HA). In the *sin3Δ* mutant, less Ste12p was found at the *MSB2* promoter (Figure 4C and 4D, compare wild type to *sin3Δ*). Msb2p and the FG pathway also control *STE12* expression. Ste12p-HA protein levels were reduced in the *sin3*Δ mutant (Figure 4E), as a result of a decrease in *STE12* expression determined by q-PCR (data not shown). Therefore, Rpd3p(L) positively regulates the FG pathway by promoting *MSB2* expression. Rpd3p(L) may also indirectly promote FG pathway activity by the regulation of other transcription factors that influence *MSB2* expression.

### Comparative Analysis of *MSB2* and *FLO11* Gene Regulation

Most of the regulators of *MSB2* expression [*MAPK, RAS, RIM101, OPI1,* and Rpd3p(L)] also regulate expression of *FLO11* [22],[66],[101], a target “hub” gene required for cell adhesion during filamentous growth and biofilm formation [8],[18],[42],[66],[102],[103],[104],[105]. Because *FLO11* is one of the most highly regulated yeast genes and a target of the FG pathway, we explored the regulation of the two genes in detail.

**Figure 5:**
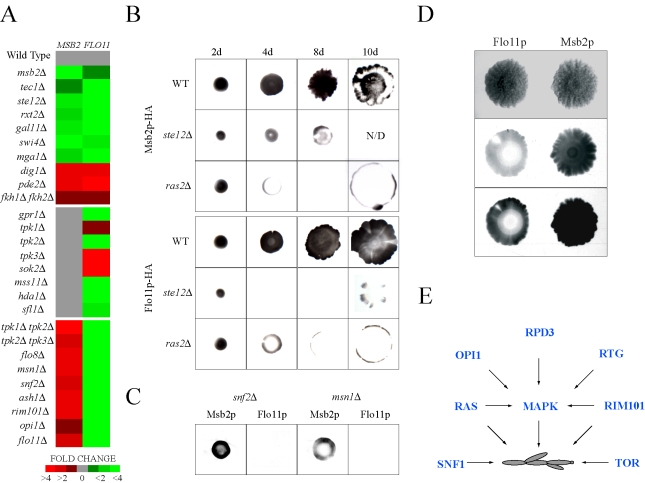
Comparative expression and secretion profiling of the *MSB2* and *FLO11* genes. (A) Quantitative PCR analysis of *MSB2* and *FLO11* expression in the indicated mutants, all of which were created in the Σ1278b background (Table S1). Heat map shows fold change in gene expression. Red, fold induced; green, fold repressed. See Table S7 for details. (B) Comparison of Msb2p-HA and Flo11p-HA secretion in microbial mats. Mats expressing Msb2p-HA (wild type, PC999; *ras2Δ*, PC2689; *ste12Δ,* PC2691) or Flo11p-HA (wild type, PC2043; *ras2Δ*, PC2693; *ste12Δ*, PC2695) were spotted onto YEPD medium (0.3% agar) on nitrocellulose filters and were allowed to expand at for 2d, 4d, 8d, and 10d. Cells were washed off the filters, which were probed with antibodies against the HA epitope. Each experiment was performed in duplicate and a representative image is shown. For some panels, the intensity of the secreted protein was below the threshold for visibility in the exposure shown. N/D, not determined. Mat perimeters were at secretion boundaries. (C) Secretion of Msb2p-HA and Flo11p-HA in the *snf2Δ* and *msn1Δ* mutants grown as mats for 8d. The experiment was performed as described in (B). (D) Comparison between Msb2p-HA and Flo11p-HA secretion on mats grown on YEP-GAL media for 8d. A light and dark exposure is shown. (E) Model for integration between pathways that control filamentous growth. The *RAS, RIM101*, and *RTG* pathways regulate the MAPK pathway. The *TOR* and *SNF1* pathways were not found to influence MAPK activity. The *RAS* [66] and *RIM101* [19] pathways likely regulate filamentous growth at multiple levels.

*MSB2* and *FLO11* expression were compared by quantitative PCR in a panel of signaling and transcription factor mutants. Phenotypic tests were used to measure FG pathway activity and Flo11p-dependent adhesion (Table S7). As expected, *MSB2* and *FLO11* expression showed similar dependencies on a number of pathways (Figure 5A, top panel). *FLO11* expression was also dependent on genes that did not affect *MSB2* expression (Figure 5A, middle panel), including primarily components of the Flo8p pathway. Unexpectedly, *FLO11* expression was also dependent on genes that inhibited *MSB2* expression, such as *snf2Δ* and *msn1Δ* (Figure 5A, bottom panel). Such inhibition might be explained by a negative-feedback loop that functions to dampen the FG pathway.

Like Msb2p, Flo11p is a secreted protein [41], which allowed an extensive comparison between the regulation of the two proteins in different conditions and genetic contexts. The levels of secreted proteins were examined in microbial mats, which expand in an Msb2p (MAPK pathway)- and Flo11p-dependent manner [105]. Msb2p-HA and Flo11p-HA showed similar dependencies on Ras2p and the FG pathway transcription factor Ste12p (Figure 5B). A “ring” pattern of Msb2p-HA and Flo11p-HA secretion was observed in the *ras2Δ* mutant (Figure 5B), which may indicate that Ras2p is required to sustain *MSB2* and *FLO11* expression in cells exposed to modest nutritional stress, like the interior of expanding mats. In contrast, Flo11p-HA secretion was dependent on proteins on which Msb2p-HA was not (Figure 5C; *snf2Δ* and *msn1Δ*). Msb2p-HA and Flo11p-HA showed opposing secretion patterns under some conditions in specific regions of mat interiors (Figure 5D) in agreement with the idea that the two genes are differentially regulated in some contexts. Therefore, expression, phenotypic, and secretion profiling corroborate the existence of a complex, partially overlapping regulatory circuit that governs the regulation of *MSB2* and a primary MAPK target hub gene *FLO11* (Figure S8).

## Discussion

In this study, we address the fundamental question of how different signal transduction pathways function in concert to reprogram cellular behavior. This question is relevant to understanding the complex cellular decision-making underlying many cellular responses. Most studies on signal transduction regulation focus on a single pathway, or on highly related pathways that potentially engage in cross talk [50]. Here we report one of the first attempts to gain a systems-level perspective on the global regulation of a MAPK pathway that controls a differentiation response. This effort relied on primarily genomic approaches including a new technique called secretion profiling, which measures release of a cell-surface MAPK regulatory protein. The “top-view” perspective we obtained has shown that many of the major regulatory proteins that control filamentous growth also control MAPK signaling. This finding is challenging to accept given that many of the pathways are currently viewed as separate entities. Nonetheless, this view is consistent with an emerging systems-level appreciation of pathway regulation in complex situations – cell differentiation, stem cell research, and cancer - where pathway interconnectedness drives the rationale for drug development and new therapeutic endeavors.

### New Connections between Signaling Networks

We specifically show that four major regulators of filamentous growth are also required for FG pathway signaling (Figure 5E). The connections between the *RIM101, OPI1*, and *RTG* pathways to FG pathway regulation are entirely novel. We further show that the *RAS* pathway and several other pathways [MAPK, Swi4p, Rpd3p(L)] converge on the promoter of the key upstream regulator (*MSB2*) of the FG pathway. Like other signal integration mechanisms [8],[66], the *MSB2* promoter is a target “hub” where multiple pathways converge. This regulatory point makes sense from the perspective that Msb2p levels dictate FG pathway activity [28]. Given that the FG pathway exhibits multimodality in regulation of pathway outputs [39], its precise modulation is critical to induce an appropriate response.

The regulation of the FG pathway differs from that of a related MAPK pathway, the pheromone response pathway, where secreted peptide pheromones provide the single major input into activation [5],[106],[107]. The pheromone response pathway is not regulated by the *RAS, RIM,* or *RTG* pathways and does not appear to be influenced by the Rpd3p(L) HDAC, although some target mating genes do require Rpd3p(L) for expression [100]. Both the FG and mating pathways are regulated by positive feedback [28],[65],[108],[109], presumably to provide signal amplification. Therefore, the FG pathway is distinguished in that it is highly sensitized to multiple external inputs. Because our screening approach was highly biased to identify regulators that function at the “top” of the pathway (at Msb2p), it is likely that the FG pathway may be subject to additional regulation not identified by this approach.

The networks that regulate filamentation signaling pathways appear in some cases to be redundant. For example, *RAS* regulates *FLO11* expression through Flo8p and the FG pathway (Figure 5E). *RIM101* similarly controls *FLO11* expression through Nrg1p/Nrg2p and FG regulation (Figure 5E). However, any one of the major filametation regulatory pathways when absent appears fully defective for the response. Such parallel processing of signaling networks might allow for “fine-tuning” of the differentiation response, or alternatively to synchronize all of the pathways to the global regulatory circuit. Such synchronization may coordinate different aspects of filamentous growth (cell-cell adhesion, cell cycle regulation, reorganization of polarity) into a cohesive response.

### RAS/cAMP Sensitizes MAPK Activity to Cellular Nutrient Status

Filamentous growth occurs within a narrow nutritional range [25], between high nutrient levels that support vegetative growth and limiting nutrients that force entry into stationary phase. The finding that Ras2p controls the overall levels of *MSB2* expression extends the initial connection between RAS and the FG pathway [61] in several important ways. First, the results explain how Ras2p connects to the FG pathway above Cdc42p. Second, the data provide a link between nutrition and FG pathway signaling at the level of cellular cAMP levels. Third, Ras2p does not appear to function as a component of the FG pathway. This conclusion is based on the conditional requirement for Ras2p in *MSB2* regulation, the suppression of the *ras2Δ* signaling defect by loss of *PDE2*, and the placement of Ras2p above *MSB2* in the FG pathway. Our results fit with the general idea that RAS controls a broad response to cellular stress that encompasses FG pathway regulation. Ras2p has recently been shown to regulate the MAPK pathway that controls sporulation [110], a diploid-specific starvation response. Ras2p may therefore function as a general regulator of MAPK signaling in response to nutritional stress.

### HDAC Regulation of MAPK Signaling

Examples by which MAPK pathways control target gene expression by recruitment of chromatin remodeling proteins is relatively common and include Rpd3p(L) regulation of HOG [99],[111],[112] and mating pathway [100] target genes. However, the regulation of MAPK activity through chromatin remodeling proteins provides a hierarchical mechanism for global cellular reprogramming. Regulation of FG pathway activity by Rpd3p(L) may contribute to the establishment of a differentiated state. Once activated, FG pathway activity may be sustained by Rpd3p(L) to reinforce accessibility to the *MSB2* promoter and formation of the filamentous cell type. Although the connection between Rpd3p(L) and nutrition is not entirely clear, Rpd3p(L) preferentially localizes to highly expressed genes, such as those required for anabolic processes [113]. Therefore, Rpd3p(L) may coordinate overall growth rate with the persistence of MAPK activity. Precedent for Rpd3p(L) HDACs in regulating developmental transitions comes from *Drosophila* DRpd3, which together with the Chameau HAT function as opposing cofactors of JNK/AP-1-dependent transcription during metamorphosis [114]. HDAC regulation of MAPK activity may therefore represent a general feature of MAPK regulation.

In conclusion, we have identified an unprecedented degree of regulation of a differentiation-dependent MAPK pathway by multiple regulatory proteins and pathways. Our findings open up new avenues for exploring the relationships between pathways and the extent of their cross-regulation with the ultimate goal of understanding all functionally relevant pathway interactions in a comprehensive manner.

## Materials and Methods

### Strains, Plasmids, and Microbiological Techniques

Yeast strains are described in Table S1. Plasmids are described in Table S2. Yeast and bacterial strains were manipulated by standard methods [115],[116]. PCR-based methods were used to generate gene disruptions, *GAL1* promoter fusions [117],[118], and insertion of epitope fusions [119], using auxotrophic and antibiotic resistant markers [120]. Integrations were confirmed by PCR Southern analysis and DNA sequencing. Plasmids pMSB2-GFP and pMSB2-HA have been described [31], as have plasmids pMSB2-lacZ and pMSB2^AG^-lacZ [39]. Plasmids containing FG pathway targets *KSS1, SVS1, PGU1,* and *YLR042C* fused to the *lacZ* gene were provided by C. Boone [65]. pFLO8 was provided by G. Fink [37]. Plasmid pIL30-*URA3* containing FgTy-*lacZ* was provided by B. Errede [121], and p*FRE-lacZ* was provided by H. Madhani [16]. The positions of the hemagglutinin (HA) epitope fusions were 500 amino acid residues for the Msb2p protein [31], at 1015 residues for the Flo11p protein [41], and at 1000 residues for the Hkr1p protein [39].

The single cell invasive growth assay [25] and the plate-washing assay [14] were performed to assess filamentous growth. Budding pattern was based on established methodology [122], and confirmed for some experiments by visual inspection of connected cells [25]. Halo assays were performed as described [123]. Microbial mat assays were performed as described [105] by growing cells on low-agar (0.3%) YEPD medium. Oleate medium was derived from standard synthetic medium lacking amino acids that was supplemented with 0.1% yeast extract, 0.5% potassium phosphate pH 6.0, and oleic acid (Toyko Kasei Kogyo Co. TCI) at a final concentration of 0.125% (w/v) solubilized in 0.5% Tween-20. Antimycin A, from Streptomyces sp. (Sigma-Aldrich, St. Louis, MO) was used at 3 µg/ml. Oligomycin (Sigma-Aldrich, St. Louis, MO) was used at 3 µg/ml, and rapamycin (Sigma-Aldrich) was added at 20 ng/ml. β-galactosidase assays were performed as previously described [124]. For some experiments, β-galactosidase assays were performed in 96-well format by growing cells containing the *MSB2-lacZ* reporter to saturation in synthetic medium lacking uracil (SD–URA) to maintain selection for the plasmid in 96-well plates at 30°C. Inductions were performed in duplicate, and the average of at least two independent experiments is reported. All experiments were carried out at 30°C unless otherwise indicated.

### Secretion Profiling of Msb2p

The *MAT* **a** haploid deletion collection [35] was transformed with a plasmid carrying a functional hemagglutinin (HA)-tagged *MSB2* gene (pMSB2-HA; [31]) using a high-throughput microtiter plate transformation protocol [125]. The deletion collection was manipulated with the BioMek 2000 automated workstation (Beckman-Coulter, Fullerton CA). For some experiments, a 96-fixed pinning tool (V & P Scientific, 23 VP 408) and plate replication tool (V & P Scientific, VP 381) were used. Sterilization was performed by sequential washes in 5% bleach, distilled water, 70% ethanol, and 95% ethanol. Ethanol (5 µl of 95%) was added to each transformation mix (200 µl) to increase transformation efficiency [126]. Transformants were harvested by centrifugation in 96-well plates, resuspended in 30 µl of water, and transferred to synthetic medium containing 2% glucose and lacking uracil (SD-URA) medium in Omnitrays (VWR International Inc. Bridgeport NJ). Transformants were pinned to SD-URA for 48 h. Colonies were transferred to 96-well plates containing 100 µl of water and pinned to SD-URA medium overlaid with a nitrocellulose filter (0.4 µm; HAHY08550 Millipore) and incubated for 48 h at 30°C. Filters were rinsed in distilled water to remove cells and probed by immunoblot analysis. Cross-contamination was estimated at 0.8% based on growth in blank positions; ∼93% of the collection (4554 mutants) was examined.

For the overexpression screen, a collection of ∼5,500 overexpression plasmids [36] was examined in a wild-type Σ1278b strain containing a functional *MSB2-HA* gene integrated at the *MSB2* locus under the control of its endogenous promoter (PC999). Plasmids were purified from *Escherichia coli* stocks by alkaline lysis DNA preparation in 96-well format. Plasmid DNA was transformed into PC999 using the high-throughput transformation protocol described above. Transformants were selected on SD-URA and screened by pinning to nitrocellulose filters on synthetic medium containing 2% galactose and lacking uracil (S+GAL-URA) to induce gene overexpression. Colonies were incubated for 2 days at 30°C. Filters were washed in a stream of water and probed by immunoblot analysis as described above. Candidate genes/deletions that were initially identified were confirmed by retesting. Approximately ∼35% of the deletion strains and 37% of the overexpression plasmids failed retesting and were considered false positives (Tables S3B and S4B). Comparative secretion profiling of Msb2p-HA, Hkr1p-HA, and Flo11p-HA was performed by transforming overexpression plasmids identified in the Msb2p-HA screen into strains that contain Hkr1p-HA (PC2740) and Flo11p-HA (PC2043). Transformants were pinned onto S-GAL-URA on nitrocellulose filters. Colonies were incubated for 48h, at which time filters were rinsed and evaluated by colony immunoblot analysis. The complete deletion collection transformed with pMSB2-HA (Table S3) and overexpression collection in strain PC999 (Table S4) were frozen in aliquots at −80°C and are available upon request.

### Computational Analysis

To compare levels of secreted protein between samples, spot intensity was measured and normalized to colony size. Small colonies initially scored as undersecretors and large colonies (particularly at plate corners) scored as hypersecretors were eliminated or flagged for retesting. As an additional test, standard immunoblots were performed from cells grown in liquid culture. Supernatants (S) and cell pellets (P) were separated by centrifugation. In most cases (>80%), differential secretion by colony immunoblot was reflected by an altered S/P ratio by standard immunoblot analysis. The ImageJ MicroArray Profile.jar algorithm (http://www.optinav.com/download/MicroArray_Profile.jar) created by Dr. Bob Dougherty and Dr. Wayne Rasband, commonly used for Microarray analysis, was used as a plugin for the ImageJ program. Image intensity was determined in 96-panel format by inverting the image after background subtraction. Plate-to-plate variation was normalized by dividing the average intensity of all spots with the average intensity of each plate, and this factor was applied to the intensity of each spot. Heat maps for expression and secretion profiling were generated as described [127]. Classification of genes based on process/function was determined using GO ontology terms in publicly available databases including the *Saccharomyces* genome database (http://www.yeastgenome.org/). Analysis of human mucin genes was facilitated by the Human Protein Reference Database (http://www.hprd.org/).

### DNA Microarray Analysis

DNA microarray analysis was performed as described [128],[129]. Wild type (PC538), *GAL-MSB2* (PC1083), *GAL-MSB2 ste12Δ* (PC1079), *GAL-MSB2 ras2Δ* (PC2949), *MSB2^Δ100–818^* (PC1516), *MSB2^Δ100–818^ ste12Δ* (PC1811), and *MSB2^Δ100–818^ ras2Δ* (PC2364) strains were grown in YEP-GAL for 6 h, at which point cells were examined by microscopy for the characteristic filamentation response. RNA was prepared by hot acid phenol and passage over an RNeasy column (Qiagen). Microarray construction, target labeling, and hybridization protocols were as described [130]. Sample comparisons were independently replicated at least 3 times from separate inductions. Fluoro-reverse experiments were used to identify sequence-specific dye biases. Arrays were scanned using a GenePix 4000 scanner (Axon Instruments). Image analysis was performed using GenePix Pro 3.0. Array features (i.e., spots) having low signal intensities or signals compromised by artifacts were removed from further analysis. Background subtracted Cy5/Cy3 ratios were log_2_ transformed and a Loess normalization strategy (*f*  = 0.67) was applied for each array using S-Plus (MathSoft, Cambridge, MA). Each feature where the |log_2_ (ratio)|≥0.8, the corresponding gene was considered differentially expressed.

### Microscopy

Differential-interference-contrast (DIC) and fluorescence microscopy using the FITC filter set were performed using an Axioplan 2 fluorescent microscope (Zeiss) with a PLAN-APOCHROMAT 100X/1.4 (oil) objective (N.A. 0.17). Digital images were obtained with the Axiocam MRm camera (Zeiss). Axiovision 4.4 software (Zeiss) was used for image acquisition and analysis. For Msb2p-GFP localization, cells were grown to saturation in selective medium to maintain plasmids harboring *MSB2-GFP* fusions. Cells were harvested by centrifugation and resuspended in YEPD medium for 4.5 h. Cells were harvested, washed three times in water, and visualized at 100X.

### Immunoblot Analysis

Immunoblots were performed as described [31]. To compare protein levels between strains, cells were grown to saturation in YEPD medium and subcultured into YEPD or YEP-GAL medium for 8 h. Culture volumes were adjusted to account for differences in cell number and harvested by centrifugation. Supernatant volumes were similarly adjusted. Cells were disrupted by addition of 200 µl lysis buffer (8 M Urea, 5 % SDS, 40 mM Tris-HCl pH 6.8, 0.1 M EDTA, 0.4 mg/ml Bromophenol blue and 1 % β-mercaptoethanol) and glass beads followed by vortexing for 5 minutes at the highest setting and boiling 5 min. Supernatants were examined by boiling in 1.5 volumes of lysis buffer for 5 min. For some experiments, cells were lysed in spheroplast buffer (1.2M sorbitol, 50 mM potassium phosphate pH 7.4, 1 mM MgCl, 250 ug/ml of zymolyase), and protein concentration was determined by Bradford assays (Bio-Rad, Hercules CA). Equal concentrations of protein were loaded into each lane. For some experiments, blots were stripped and re-probed using anti-actin monoclonal antibodies (Chemicon; Billerica, MA). Monoclonal antibodies against the HA epitope were used (12CA5). Proteins were separated by SDS-PAGE on 10% or 10%–20% gradient gels (Bio-Rad, Hercules CA) and transferred to nitrocellulose membranes (protran BA85, VWR International Inc. Bridgeport NJ). Membranes were incubated in blocking buffer (5% nonfat dry milk, 10 mM Tris-HCl pH 8, 150 mM NaCl and 0.05% Tween 20) for 1 hr at 25°C. Nitrocellulose membranes were incubated for 18 hr at 4°C in blocking buffer containing primary antibodies. ECL Plus immunoblotting reagents were used to detect secondary antibodies (Amersham Biosciences, Piscataway NJ). Immunoblots of proteins secreted from mats were performed by growing cells on nitrocellulose filters on low-agar (0.3%) YEPD medium.

### Chromatin Immunoprecipitation (ChIP) Analysis

ChIP assays were performed as described [131]. Strains PC3021 (*STE12-HA*), PC3353 (*sin3Δ, STE12-HA*) and PC3579 (*SIN3-HA*) were grown in YEPD medium for 8 h. Cross-linking was performed with 1% formaldehyde for 15 min at 25°C. Cells were collected by centrifugation and washed twice in PBS buffer. Cells were resuspended in ChIP lysis buffer (Upstate, Billerica, CA), and lysed by Fast Prep 24 (MP) for one cycle at 6.5 for 45 sec. After puncturing the bottom of Fast Prep tubes with a 22 gauge needle, lysates were collected by centrifugation. DNA was sheared by sonication on a Branson Digital Sonifier at setting 20% amplitude, 15 pulses for 20 sec, 55 sec. rest. Pull downs were performed using a ChIP assay kit (Upstate) with anti-HA antibodies with 10% input sample set aside for a control. qPCR was used to determine relative amount of immunoprecipitated specific DNA loci in IP, Input, and Mock (no antibody) samples. The housekeeping *ACT1* gene was used to normalize quantification in qPCR reactions. Data are expressed as IP/Input where ΔΔCt = (Ct IP_MSB2-Ct IP_ACT1)-(Ct Input_MSB2-Ct Input_ACT1). Primers used were *MSB2* promoter forward 5′-CGATAGCTGATAGACTGTGGAGTCG-3′ and reverse 5′- CTGGCAACGCCCGACGTGTCTAGCC-3′, *MSB2* intergenic region forward 5′- TGACCAAACTTCGACTGCTGG-3′ and reverse 5′- AGCTGCTGATGCAGTGGTAA-3′; and *ACT1* forward 5′- GGCTTCTTTGACTACCTTCCAACA-3′ and reverse 5′- GATGGACCACTTTCGTCGTATTC-3′. ChIP pull downs were also visualized by gel electrophoresis.

### mRNA Level Determination Using Quantitative PCR

Total RNA was isolated from 25 ml cultures grown in YEP GAL for 8 h using hot acid phenol extraction. cDNA synthesis was carried out using 1 µg RNA and the iScript cDNA Synthesis Kit (Bio-Rad; Hercules CA) according to the manufacturer's instructions. One tenth of the synthesized cDNA was used as the template for real-time PCR. 25 ul real time PCR reactions were performed on the BioRad MyiQ Cycler with iQ SYBR Green Supermix (Bio-Rad). RT qPCR was performed using the following amplification cycles: initial denaturation for 8 min at 95°C, followed by 35×cycle 2 (denaturation for 15 sec at 95°C and annealing for 1 min at 60°C). Melt curve data collection was enabled by decreasing the set point temperature after cycle 2 by 0.5°C. The specificity of amplicons was confirmed by generating the melt curve profile of all amplified products. Gene expression was quantified as described [132]. Primers were based on a previous report [133] and were *FLO11* forward 5′- GTTCAACCAGTCCAAGCGAAA-3′ and reverse 5′- GTAGTTACAGGTGTGGTAGGTGAAGTG-3′and those described for ChIP assays above. All reactions were performed in duplicate and average values are reported.

## Supporting Information

Figure S1 Genes that regulate Msb2p-HA secretion comprise a number of different functional categories. Pie charts of functional categories enriched in the genomic screens. Far left, functional categories that contributed to Msb2p-HA secretion; middle panel, functional categories that were inhibitory for Msb2p-HA secretion; far right, the overall functional classification of genes in the yeast genome for reference. Functional classification of yeast genes was facilitated by *SGD* (http://www.yeastgenome.org/).
(6.08 MB TIF)

Figure S2 *MSB2* expression is influenced by a different extracellular stimuli. The expression of an *MSB2-lacZ* fusion was examined under the conditions described. Cells were grown to mid-log phase in YEPD medium (∼6h) or medium lacking glucose (YEP), containing a poor carbon source (YEP-GAL), or to saturation in a poor carbon source (YEP-GAL 16h). *MSB2-lacZ* expression was also compared in medium supplemented with the amino acid glutamate and medium at pH 3, pH 7, and pH 8. Cells were harvested, and β-galactosidase assays were performed in independent replicates. The error bars represent standard deviation between experiments.
(2.70 MB TIF)

Figure S3 Genetic suppression analysis of *RAS* pathway components based on the activity of the *FUS1-HIS3* reporter. (A) The morphology of wild-type cells and cells containing the activated allele *MSB2^Δ100–818^* or overexpressing *MSB2* (*GAL-MSB2*) in combination with *ras2Δ* and *ste12Δ* mutations. Images were taken by DIC at 100X. Bar, 20 microns. (B) Equal concentrations of cells of the indicated genotypes were spotted onto synthetic medium supplemented with glucose (SD) or galactose (S+GAL) that contained all amino acids (+AA), or that lacked uracil (-URA), and/or lacking histidine (-HIS). Plates were incubated at 30°C and spots were photographed. Growth on medium is indicative of the activity of the FG pathway. In the top panel, *SHO1^P120L^* and *SHO1^S22oF^* bypass the signaling defect of the *ras2Δ* mutant suggesting that Sho1p functions below Ras2p, and in line with the idea that Ras2p controls *MSB2* expression. In the middle panel, overexpression of *MSB2* bypasses the signaling defect of the *ras2Δ* mutant to a greater degree than the activated allele *MSB2^Δ100–818^*. In the bottom panel, Flo8p pathway mutants are not required to activate the FG pathway reporter.
(3.55 MB TIF)

Figure S4 The FG pathway does not regulate RAS pathway outputs. (A) The *ras2Δ* mutant accumulates glycogen, whereas FG pathway mutants do not. Equal concentrations of cells of the indicated genotypes were spotted onto YEPD medium for 4d at 30°C. Plates were exposed to iodine vapor for ∼1 min and photographed. (B) Chronological survival of strains lacking Ras2p or FG MAPK components. Longevity was evaluated in YEPD medium over a 21-day time course. At the indicated days, a sample of the culture was removed and examined for the number of viable colonies by serial dilution on YEPD medium. (C) Sensitivity of RAS and MAPK components to oxidative stress. Equal concentrations of cells were spotted onto YEPD and YEP-GAL media containing the indicated volume of hydrogen peroxide. Plates were incubated for 2d at 30°C and photographed. (D) The *ras2* mutant is resistant to oleate in comparison to wild-type cells and FG pathway mutants. Equal concentrations of cells were spotted onto media containing oleate and examined after 7d at 30°C.
(2.72 MB TIF)

Figure S5 Genetic analysis of the Sin3p-Rpd3p complex in regulating MAPK signaling. (A) Halo assays. Wild-type cells and the indicated mutants were spread onto YEPD medium and 1 µl of 1uM α-factor was applied to plates. (B) Genetic suppression analysis of the *rxt2Δ* mutant in combination with other mutants in the filamentous growth pathway. (C) Cell morphologies of the *ras2Δ* and *rxt2Δ* mutants in combination with the activated allele *MSB2^Δ100–818^*. Bar, 10 microns.
(4.03 MB TIF)

Figure S6 The Dig1p protein does not function through Rpd3(L). (A) Plate-washing assay showing the agar-invasion defects of Sin3p-Rpd3p complex mutants. (B) Immunoblot analysis of Msb2p-HA levels in wild-type cells and the *dig1Δ rxt2Δ* and *dig1Δrxt2Δ* double mutant.
(9.03 MB TIF)

Figure S7 The relationship between filamentous growth regulation and cellular respiration. (A) Secretion profiling identifies nutritional regulatory and enzymatic genes and genes that function in respiration or mitochondrial functions. (B) The role of the mitochondria on filamentous growth. Antimycin and oligomycin, but not rapamycin induce filamentation. Petite mutants are capable of undergoing filamentous growth. Cells were grown on semi-solid agar medium for 24 h and assessed by microscopy for filamentous growth. Bar, 10 microns. (C) A functional mitochondria is required for mat expansion. Mats were incubated on YEPD medium and assessed over time for expansion. On the final day, the plate was washed to reveal invaded cells. (D) Expression of *MSB2-lacZ* in cells exposed to mitochondrial inhibitors antimycin or oligomycin or in peitie mutants.
(11.14 MB TIF)

Figure S8 Model for the combinatorial regulation of the *MSB2* and *FLO11* promoters. Nutritional information is conveyed to the regulation of *MSB2* expression through Ras2p/cAMP and Rpd3(L). Rpd3(L) is required *MSB2* expression by association with the *MSB2* promoter (blue line). Ras2p contributes to MAPK regulation by several mechanisms (red lines). Ras2p/cAMP is required to activate *MSB2* expression through an unknown mechanism (denoted by question mark). In contrast, Ras2p-cAMP-PKA-Flo8p is required for *FLO11* expression through the Flo8p transcription factor. Msb2p regulates *FLO11* expression by MAPK signaling (green arrows). Msb2p further regulates its own expression through autofeedback by the MAPK pathway (green arrows). The dotted green line represents the feed-forward loop, by MAPK induction of genes that function in nutritional scavenging. *FLO11*-specific regulators are shown in yellow (Snf1p, TOR).
(4.19 MB TIF)

Table S1 Yeast strains.
(0.22 MB DOC)

Table S2 Plasmids used in this study.
(0.08 MB DOC)

Table S3 Msb2p-HA secretion analysis in the *MAT*a collection of deletion mutants.
(0.71 MB XLS)

Table S4 Genes that when overexpressed influence the secretion of Msb2p-HA.
(1.33 MB XLS)

Table S5 Secondary screens to evaluate Msb2p-HA secretion.
(0.06 MB XLS)

Table S6 Expression profiling of cells containing an activated allele of *MSB2* or overexpressing the *MSB2* gene in combination with *ras2* and *ste12*.
(8.71 MB XLS)

Table S7 Comparison of *MSB2* and *FLO11* expression levels, invasive growth, and MAPK signaling in mutants identified by secretion profiling.
(0.03 MB XLS)

Table S8 Comparative secretion profiling between yeast mucins.
(0.16 MB XLS)

## References

[1] Doroquez DB, Rebay I (2006). Signal integration during development: mechanisms of EGFR and Notch pathway function and cross-talk.. Crit Rev Biochem Mol Biol.

[2] Hurlbut GD, Kankel MW, Lake RJ, Artavanis-Tsakonas S (2007). Crossing paths with Notch in the hyper-network.. Curr Opin Cell Biol.

[3] Wagner EF, Nebreda AR (2009). Signal integration by JNK and p38 MAPK pathways in cancer development.. Nat Rev Cancer.

[4] Gimeno CJ, Ljungdahl PO, Styles CA, Fink GR (1992). Unipolar cell divisions in the yeast S. cerevisiae lead to filamentous growth: regulation by starvation and RAS.. Cell.

[5] Schwartz MA, Madhani HD (2004). Principles of map kinase signaling specificity in Saccharomyces cerevisiae.. Annu Rev Genet.

[6] Verstrepen KJ, Klis FM (2006). Flocculation, adhesion and biofilm formation in yeasts.. Mol Microbiol.

[7] Jin R, Dobry CJ, McCown PJ, Kumar A (2008). Large-scale analysis of yeast filamentous growth by systematic gene disruption and overexpression.. Mol Biol Cell.

[8] Borneman AR, Leigh-Bell JA, Yu H, Bertone P, Gerstein M (2006). Target hub proteins serve as master regulators of development in yeast.. Genes Dev.

[9] Prinz S, Avila-Campillo I, Aldridge C, Srinivasan A, Dimitrov K (2004). Control of yeast filamentous-form growth by modules in an integrated molecular network.. Genome Res.

[10] Madhani HD, Galitski T, Lander ES, Fink GR (1999). Effectors of a developmental mitogen-activated protein kinase cascade revealed by expression signatures of signaling mutants.. Proc Natl Acad Sci U S A.

[11] Lo HJ, Kohler JR, DiDomenico B, Loebenberg D, Cacciapuoti A (1997). Nonfilamentous C. albicans mutants are avirulent.. Cell.

[12] Whiteway M, Bachewich C (2007). Morphogenesis in Candida albicans.. Annu Rev Microbiol.

[13] Nobile CJ, Mitchell AP (2006). Genetics and genomics of Candida albicans biofilm formation.. Cell Microbiol.

[14] Roberts RL, Fink GR (1994). Elements of a single MAP kinase cascade in Saccharomyces cerevisiae mediate two developmental programs in the same cell type: mating and invasive growth.. Genes Dev.

[15] Madhani HD, Fink GR (1997). Combinatorial control required for the specificity of yeast MAPK signaling.. Science.

[16] Madhani HD, Styles CA, Fink GR (1997). MAP kinases with distinct inhibitory functions impart signaling specificity during yeast differentiation.. Cell.

[17] Mosch HU, Kubler E, Krappmann S, Fink GR, Braus GH (1999). Crosstalk between the Ras2p-controlled mitogen-activated protein kinase and cAMP pathways during invasive growth of Saccharomyces cerevisiae.. Mol Biol Cell.

[18] Vinod PK, Sengupta N, Bhat PJ, Venkatesh KV (2008). Integration of global signaling pathways, cAMP-PKA, MAPK and TOR in the regulation of FLO11.. PLoS ONE.

[19] Lamb TM, Mitchell AP (2003). The transcription factor Rim101p governs ion tolerance and cell differentiation by direct repression of the regulatory genes NRG1 and SMP1 in Saccharomyces cerevisiae.. Mol Cell Biol.

[20] Barwell KJ, Boysen JH, Xu W, Mitchell AP (2005). Relationship of DFG16 to the Rim101p pH response pathway in Saccharomyces cerevisiae and Candida albicans.. Eukaryot Cell.

[21] Rothfels K, Tanny JC, Molnar E, Friesen H, Commisso C (2005). Components of the ESCRT pathway, DFG16, and YGR122w are required for Rim101 to act as a corepressor with Nrg1 at the negative regulatory element of the DIT1 gene of Saccharomyces cerevisiae.. Mol Cell Biol.

[22] Reynolds TB (2006). The Opi1p transcription factor affects expression of FLO11, mat formation, and invasive growth in Saccharomyces cerevisiae.. Eukaryot Cell.

[23] Kuchin S, Vyas VK, Carlson M (2002). Snf1 protein kinase and the repressors Nrg1 and Nrg2 regulate FLO11, haploid invasive growth, and diploid pseudohyphal differentiation.. Mol Cell Biol.

[24] Kuchin S, Vyas VK, Carlson M (2003). Role of the yeast Snf1 protein kinase in invasive growth.. Biochem Soc Trans.

[25] Cullen PJ, Sprague GF (2000). Glucose depletion causes haploid invasive growth in yeast.. Proc Natl Acad Sci U S A.

[26] Palecek SP, Parikh AS, Kron SJ (2000). Genetic analysis reveals that FLO11 upregulation and cell polarization independently regulate invasive growth in Saccharomyces cerevisiae.. Genetics.

[27] Mosch HU, Fink GR (1997). Dissection of filamentous growth by transposon mutagenesis in Saccharomyces cerevisiae.. Genetics.

[28] Cullen PJ, Sabbagh W, Graham E, Irick MM, van Olden EK (2004). A signaling mucin at the head of the Cdc42- and MAPK-dependent filamentous growth pathway in yeast.. Genes Dev.

[29] Singh PK, Hollingsworth MA (2006). Cell surface-associated mucins in signal transduction.. Trends Cell Biol.

[30] Park HO, Bi E (2007). Central roles of small GTPases in the development of cell polarity in yeast and beyond.. Microbiol Mol Biol Rev.

[31] Vadaie N, Dionne H, Akajagbor DS, Nickerson SR, Krysan DJ (2008). Cleavage of the signaling mucin Msb2 by the aspartyl protease Yps1 is required for MAPK activation in yeast.. J Cell Biol.

[32] Roberts CJ, Raymond CK, Yamashiro CT, Stevens TH (1991). Methods for studying the yeast vacuole.. Methods Enzymol.

[33] Schluter C, Lam KK, Brumm J, Wu BW, Saunders M (2008). Global Analysis of Yeast Endosomal Transport Identifies the Vps55/68 Sorting Complex.. Mol Biol Cell.

[34] Bonangelino CJ, Chavez EM, Bonifacino JS (2002). Genomic screen for vacuolar protein sorting genes in Saccharomyces cerevisiae.. Mol Biol Cell.

[35] Winzeler EA, Shoemaker DD, Astromoff A, Liang H, Anderson K (1999). Functional characterization of the S. cerevisiae genome by gene deletion and parallel analysis.. Science.

[36] Gelperin DM, White MA, Wilkinson ML, Kon Y, Kung LA (2005). Biochemical and genetic analysis of the yeast proteome with a movable ORF collection.. Genes Dev.

[37] Liu H, Styles CA, Fink GR (1996). Saccharomyces cerevisiae S288C has a mutation in FLO8, a gene required for filamentous growth.. Genetics.

[38] Niu W, Li Z, Zhan W, Iyer VR, Marcotte EM (2008). Mechanisms of cell cycle control revealed by a systematic and quantitative overexpression screen in S. cerevisiae.. PLoS Genet.

[39] Pitoniak A, Birkaya B, Dionne HS, Vadiae N, Cullen PJ (2009). The Signaling Mucins Msb2 and Hkr1 Differentially Regulate the Filamentation MAPK Pathway and Contribute to a Multimodal Response.. Mol Biol Cell.

[40] Tatebayashi K, Tanaka K, Yang HY, Yamamoto K, Matsushita Y (2007). Transmembrane mucins Hkr1 and Msb2 are putative osmosensors in the SHO1 branch of yeast HOG pathway.. Embo J.

[41] Karunanithi SR, Vadaie N, Birkaya B, Dionne HM, Joshi J (SUBMITTED) Regulation and Functional Basis of Mucin Shedding in a Unicellular Eukaryote..

[42] Guo B, Styles CA, Feng Q, Fink GR (2000). A Saccharomyces gene family involved in invasive growth, cell-cell adhesion, and mating.. Proc Natl Acad Sci U S A.

[43] Krysan DJ, Ting EL, Abeijon C, Kroos L, Fuller RS (2005). Yapsins are a family of aspartyl proteases required for cell wall integrity in Saccharomyces cerevisiae.. Eukaryot Cell.

[44] Andrews BJ, Herskowitz I (1989). The yeast SWI4 protein contains a motif present in developmental regulators and is part of a complex involved in cell-cycle-dependent transcription.. Nature.

[45] Breeden L, Mikesell GE (1991). Cell cycle-specific expression of the SWI4 transcription factor is required for the cell cycle regulation of HO transcription.. Genes Dev.

[46] Nasmyth K, Dirick L (1991). The role of SWI4 and SWI6 in the activity of G1 cyclins in yeast.. Cell.

[47] Ogas J, Andrews BJ, Herskowitz I (1991). Transcriptional activation of CLN1, CLN2, and a putative new G1 cyclin (HCS26) by SWI4, a positive regulator of G1-specific transcription.. Cell.

[48] Baetz K, Andrews B (1999). Regulation of cell cycle transcription factor Swi4 through auto-inhibition of DNA binding.. Mol Cell Biol.

[49] Bean JM, Siggia ED, Cross FR (2005). High functional overlap between MluI cell-cycle box binding factor and Swi4/6 cell-cycle box binding factor in the G1/S transcriptional program in Saccharomyces cerevisiae.. Genetics.

[50] Bardwell L (2006). Mechanisms of MAPK signalling specificity.. Biochem Soc Trans.

[51] Abdullah U, Cullen PJ (2009). The tRNA modification complex elongator regulates the Cdc42-dependent mitogen-activated protein kinase pathway that controls filamentous growth in yeast.. Eukaryot Cell.

[52] Jia Y, Rothermel B, Thornton J, Butow RA (1997). A basic helix-loop-helix-leucine zipper transcription complex in yeast functions in a signaling pathway from mitochondria to the nucleus.. Mol Cell Biol.

[53] Dilova I, Aronova S, Chen JC, Powers T (2004). Tor signaling and nutrient-based signals converge on Mks1p phosphorylation to regulate expression of Rtg1.Rtg3p-dependent target genes.. J Biol Chem.

[54] Ferreira Junior JR, Spirek M, Liu Z, Butow RA (2005). Interaction between Rtg2p and Mks1p in the regulation of the RTG pathway of Saccharomyces cerevisiae.. Gene.

[55] Liu Z, Sekito T, Epstein CB, Butow RA (2001). RTG-dependent mitochondria to nucleus signaling is negatively regulated by the seven WD-repeat protein Lst8p.. Embo J.

[56] Giannattasio S, Liu Z, Thornton J, Butow RA (2005). Retrograde response to mitochondrial dysfunction is separable from TOR1/2 regulation of retrograde gene expression.. J Biol Chem.

[57] Klig LS, Homann MJ, Carman GM, Henry SA (1985). Coordinate regulation of phospholipid biosynthesis in Saccharomyces cerevisiae: pleiotropically constitutive opi1 mutant.. J Bacteriol.

[58] White MJ, Hirsch JP, Henry SA (1991). The OPI1 gene of Saccharomyces cerevisiae, a negative regulator of phospholipid biosynthesis, encodes a protein containing polyglutamine tracts and a leucine zipper.. J Biol Chem.

[59] Voth WP, Yu Y, Takahata S, Kretschmann KL, Lieb JD (2007). Forkhead proteins control the outcome of transcription factor binding by antiactivation.. Embo J.

[60] Kim TS, Lee SB, Kang HS (2004). Glucose repression of STA1 expression is mediated by the Nrg1 and Sfl1 repressors and the Srb8-11 complex.. Mol Cell Biol.

[61] Mosch HU, Roberts RL, Fink GR (1996). Ras2 signals via the Cdc42/Ste20/mitogen-activated protein kinase module to induce filamentous growth in Saccharomyces cerevisiae.. Proc Natl Acad Sci U S A.

[62] Carter GW, Rupp S, Fink GR, Galitski T (2006). Disentangling information flow in the Ras-cAMP signaling network.. Genome Res.

[63] Robertson LS, Causton HC, Young RA, Fink GR (2000). The yeast A kinases differentially regulate iron uptake and respiratory function.. Proc Natl Acad Sci U S A.

[64] Zaman S, Lippman SI, Schneper L, Slonim N, Broach JR (2009). Glucose regulates transcription in yeast through a network of signaling pathways.. Mol Syst Biol.

[65] Roberts CJ, Nelson B, Marton MJ, Stoughton R, Meyer MR (2000). Signaling and circuitry of multiple MAPK pathways revealed by a matrix of global gene expression profiles.. Science.

[66] Rupp S, Summers E, Lo HJ, Madhani H, Fink G (1999). MAP kinase and cAMP filamentation signaling pathways converge on the unusually large promoter of the yeast FLO11 gene.. Embo J.

[67] Zaman S, Lippman SI, Zhao X, Broach JR (2008). How Saccharomyces Responds to Nutrients..

[68] Santangelo GM (2006). Glucose signaling in Saccharomyces cerevisiae.. Microbiol Mol Biol Rev.

[69] Boy-Marcotte E, Ikonomi P, Jacquet M (1996). SDC25, a dispensable Ras guanine nucleotide exchange factor of Saccharomyces cerevisiae differs from CDC25 by its regulation.. Mol Biol Cell.

[70] Poullet P, Crechet JB, Bernardi A, Parmeggiani A (1995). Properties of the catalytic domain of sdc25p, a yeast GDP/GTP exchange factor of Ras proteins. Complexation with wild-type Ras2p, [S24N]Ras2p and [R80D, N81D]Ras2p.. Eur J Biochem.

[71] Toda T, Uno I, Ishikawa T, Powers S, Kataoka T (1985). In yeast, RAS proteins are controlling elements of adenylate cyclase.. Cell.

[72] Sass P, Field J, Nikawa J, Toda T, Wigler M (1986). Cloning and characterization of the high-affinity cAMP phosphodiesterase of Saccharomyces cerevisiae.. Proc Natl Acad Sci U S A.

[73] Pan X, Heitman J (1999). Cyclic AMP-dependent protein kinase regulates pseudohyphal differentiation in Saccharomyces cerevisiae.. Mol Cell Biol.

[74] van Dyk D, Pretorius IS, Bauer FF (2005). Mss11p is a central element of the regulatory network that controls FLO11 expression and invasive growth in Saccharomyces cerevisiae.. Genetics.

[75] Robertson LS, Fink GR (1998). The three yeast A kinases have specific signaling functions in pseudohyphal growth.. Proc Natl Acad Sci U S A.

[76] Lorenz MC, Pan X, Harashima T, Cardenas ME, Xue Y (2000). The G protein-coupled receptor gpr1 is a nutrient sensor that regulates pseudohyphal differentiation in Saccharomyces cerevisiae.. Genetics.

[77] Lemaire K, Van de Velde S, Van Dijck P, Thevelein JM (2004). Glucose and Sucrose Act as Agonist and Mannose as Antagonist Ligands of the G Protein-Coupled Receptor Gpr1 in the Yeast Saccharomyces cerevisiae.. Mol Cell.

[78] Harashima T, Anderson S, Yates JR, Heitman J (2006). The kelch proteins Gpb1 and Gpb2 inhibit Ras activity via association with the yeast RasGAP neurofibromin homologs Ira1 and Ira2.. Mol Cell.

[79] Peeters T, Louwet W, Gelade R, Nauwelaers D, Thevelein JM (2006). Kelch-repeat proteins interacting with the Galpha protein Gpa2 bypass adenylate cyclase for direct regulation of protein kinase A in yeast.. Proc Natl Acad Sci U S A.

[80] Harashima T, Heitman J (2002). The Galpha protein Gpa2 controls yeast differentiation by interacting with kelch repeat proteins that mimic Gbeta subunits.. Mol Cell.

[81] Broach JR (1991). RAS genes in Saccharomyces cerevisiae: signal transduction in search of a pathway.. Trends Genet.

[82] Charizanis C, Juhnke H, Krems B, Entian KD (1999). The oxidative stress response mediated via Pos9/Skn7 is negatively regulated by the Ras/PKA pathway in Saccharomyces cerevisiae.. Mol Gen Genet.

[83] Hasan R, Leroy C, Isnard AD, Labarre J, Boy-Marcotte E (2002). The control of the yeast H2O2 response by the Msn2/4 transcription factors.. Mol Microbiol.

[84] Fabrizio P, Liou LL, Moy VN, Diaspro A, Valentine JS (2003). SOD2 functions downstream of Sch9 to extend longevity in yeast.. Genetics.

[85] Longo VD, Ellerby LM, Bredesen DE, Valentine JS, Gralla EB (1997). Human Bcl-2 reverses survival defects in yeast lacking superoxide dismutase and delays death of wild-type yeast.. J Cell Biol.

[86] Sinclair D, Mills K, Guarente L (1998). Aging in Saccharomyces cerevisiae.. Annu Rev Microbiol.

[87] Igual JC, Navarro B (1996). Respiration and low cAMP-dependent protein kinase activity are required for high-level expression of the peroxisomal thiolase gene in Saccharomyces cerevisiae.. Mol Gen Genet.

[88] Rundlett SE, Carmen AA, Kobayashi R, Bavykin S, Turner BM (1996). HDA1 and RPD3 are members of distinct yeast histone deacetylase complexes that regulate silencing and transcription.. Proc Natl Acad Sci U S A.

[89] Kadosh D, Struhl K (1997). Repression by Ume6 involves recruitment of a complex containing Sin3 corepressor and Rpd3 histone deacetylase to target promoters.. Cell.

[90] Kasten MM, Dorland S, Stillman DJ (1997). A large protein complex containing the yeast Sin3p and Rpd3p transcriptional regulators.. Mol Cell Biol.

[91] Lechner T, Carrozza MJ, Yu Y, Grant PA, Eberharter A (2000). Sds3 (suppressor of defective silencing 3) is an integral component of the yeast Sin3[middle dot]Rpd3 histone deacetylase complex and is required for histone deacetylase activity.. J Biol Chem.

[92] Carrozza MJ, Li B, Florens L, Suganuma T, Swanson SK (2005). Histone H3 methylation by Set2 directs deacetylation of coding regions by Rpd3S to suppress spurious intragenic transcription.. Cell.

[93] Colina AR, Young D (2005). Raf60, a novel component of the Rpd3 histone deacetylase complex required for Rpd3 activity in Saccharomyces cerevisiae.. J Biol Chem.

[94] Qi M, Elion EA (2005). MAP kinase pathways.. J Cell Sci.

[95] Murphy LO, Blenis J (2006). MAPK signal specificity: the right place at the right time.. Trends Biochem Sci.

[96] Cook JG, Bardwell L, Kron SJ, Thorner J (1996). Two novel targets of the MAP kinase Kss1 are negative regulators of invasive growth in the yeast Saccharomyces cerevisiae.. Genes Dev.

[97] Olson KA, Nelson C, Tai G, Hung W, Yong C (2000). Two regulators of Ste12p inhibit pheromone-responsive transcription by separate mechanisms.. Mol Cell Biol.

[98] Chou S, Lane S, Liu H (2006). Regulation of mating and filamentation genes by two distinct Ste12 complexes in Saccharomyces cerevisiae.. Mol Cell Biol.

[99] De Nadal E, Zapater M, Alepuz PM, Sumoy L, Mas G (2004). The MAPK Hog1 recruits Rpd3 histone deacetylase to activate osmoresponsive genes.. Nature.

[100] Vidal M, Strich R, Esposito RE, Gaber RF (1991). RPD1 (SIN3/UME4) is required for maximal activation and repression of diverse yeast genes.. Mol Cell Biol.

[101] Barrales RR, Jimenez J, Ibeas JI (2008). Identification of novel activation mechanisms for FLO11 regulation in Saccharomyces cerevisiae.. Genetics.

[102] Lo WS, Dranginis AM (1996). FLO11, a yeast gene related to the STA genes, encodes a novel cell surface flocculin.. J Bacteriol.

[103] Lambrechts MG, Bauer FF, Marmur J, Pretorius IS (1996). Muc1, a mucin-like protein that is regulated by Mss10, is critical for pseudohyphal differentiation in yeast.. Proc Natl Acad Sci U S A.

[104] Lo WS, Dranginis AM (1998). The cell surface flocculin Flo11 is required for pseudohyphae formation and invasion by Saccharomyces cerevisiae.. Mol Biol Cell.

[105] Reynolds TB, Fink GR (2001). Bakers' yeast, a model for fungal biofilm formation.. Science.

[106] Bardwell L (2004). A walk-through of the yeast mating pheromone response pathway.. Peptides.

[107] Elion EA (2000). Pheromone response, mating and cell biology.. Curr Opin Microbiol.

[108] Nakayama N, Miyajima A, Arai K (1987). Common signal transduction system shared by STE2 and STE3 in haploid cells of Saccharomyces cerevisiae: autocrine cell-cycle arrest results from forced expression of STE2.. Embo J.

[109] Bender A, Sprague GF (1986). Yeast peptide pheromones, a-factor and alpha-factor, activate a common response mechanism in their target cells.. Cell.

[110] McDonald CM, Wagner M, Dunham MJ, Shin ME, Ahmed NT (2009). The Ras/cAMP pathway and the CDK-like kinase Ime2 regulate the MAPK Smk1 and spore morphogenesis in Saccharomyces cerevisiae.. Genetics.

[111] Mas G, de Nadal E, Dechant R, de la Concepcion ML, Logie C (2009). Recruitment of a chromatin remodelling complex by the Hog1 MAP kinase to stress genes.. Embo J.

[112] Dunn KL, Espino PS, Drobic B, He S, Davie JR (2005). The Ras-MAPK signal transduction pathway, cancer and chromatin remodeling.. Biochem Cell Biol.

[113] Kurdistani SK, Robyr D, Tavazoie S, Grunstein M (2002). Genome-wide binding map of the histone deacetylase Rpd3 in yeast.. Nat Genet.

[114] Miotto B, Sagnier T, Berenger H, Bohmann D, Pradel J (2006). Chameau HAT and DRpd3 HDAC function as antagonistic cofactors of JNK/AP-1-dependent transcription during Drosophila metamorphosis.. Genes Dev.

[115] Sambrook J, Fritsch EF, Maniatis T (1989). Molecular cloning: a laboratory manual..

[116] Rose MD, Winston F, Hieter P (1990). Methods in yeast genetics..

[117] Baudin A, Ozier-Kalogeropoulos O, Denouel A, Lacroute F, Cullin C (1993). A simple and efficient method for direct gene deletion in Saccharomyces cerevisiae.. Nucleic Acids Res.

[118] Longtine MS, McKenzie A, Demarini DJ, Shah NG, Wach A (1998). Additional modules for versatile and economical PCR-based gene deletion and modification in Saccharomyces cerevisiae.. Yeast.

[119] Schneider BL, Seufert W, Steiner B, Yang QH, Futcher AB (1995). Use of polymerase chain reaction epitope tagging for protein tagging in Saccharomyces cerevisiae.. Yeast.

[120] Goldstein AL, McCusker JH (1999). Three new dominant drug resistance cassettes for gene disruption in Saccharomyces cerevisiae.. Yeast.

[121] Laloux I, Jacobs E, Dubois E (1994). Involvement of SRE element of Ty1 transposon in TEC1-dependent transcriptional activation.. Nucleic Acids Res.

[122] Chant J, Pringle JR (1995). Patterns of bud-site selection in the yeast Saccharomyces cerevisiae.. J Cell Biol.

[123] Jenness DD, Goldman BS, Hartwell LH (1987). Saccharomyces cerevisiae mutants unresponsive to alpha-factor pheromone: alpha-factor binding and extragenic suppression.. Mol Cell Biol.

[124] Cullen PJ, Schultz J, Horecka J, Stevenson BJ, Jigami Y (2000). Defects in protein glycosylation cause SHO1-dependent activation of a STE12 signaling pathway in yeast.. Genetics.

[125] Gietz RD, Schiestl RH (2007). Microtiter plate transformation using the LiAc/SS carrier DNA/PEG method.. Nat Protoc.

[126] Gietz RD, Woods RA (2002). Transformation of yeast by lithium acetate/single-stranded carrier DNA/polyethylene glycol method.. Methods Enzymol.

[127] Eisen MB, Spellman PT, Brown PO, Botstein D (1998). Cluster analysis and display of genome-wide expression patterns.. Proc Natl Acad Sci U S A.

[128] DeRisi JL, Iyer VR, Brown PO (1997). Exploring the metabolic and genetic control of gene expression on a genomic scale.. Science.

[129] Lashkari DA, DeRisi JL, McCusker JH, Namath AF, Gentile C (1997). Yeast microarrays for genome wide parallel genetic and gene expression analysis.. Proc Natl Acad Sci U S A.

[130] Fazzio TG, Kooperberg C, Goldmark JP, Neal C, Basom R (2001). Widespread collaboration of Isw2 and Sin3-Rpd3 chromatin remodeling complexes in transcriptional repression.. Mol Cell Biol.

[131] Yu MC, Lamming DW, Eskin JA, Sinclair DA, Silver PA (2006). The role of protein arginine methylation in the formation of silent chromatin.. Genes Dev.

[132] Pfaffl MW (2001). A new mathematical model for relative quantification in real-time RT-PCR.. Nucleic Acids Res.

[133] Voynov V, Verstrepen KJ, Jansen A, Runner VM, Buratowski S (2006). Genes with internal repeats require the THO complex for transcription.. Proc Natl Acad Sci U S A.

[134] McCaffrey G, Clay FJ, Kelsay K, Sprague GF (1987). Identification and regulation of a gene required for cell fusion during mating of the yeast Saccharomyces cerevisiae.. Mol Cell Biol.

[135] O'Rourke SM, Herskowitz I (2004). Unique and redundant roles for HOG MAPK pathway components as revealed by whole-genome expression analysis.. Mol Biol Cell.

[136] Rivers DM, Sprague GF (2003). Autocrine activation of the pheromone response pathway in matalpha2- cells is attenuated by SST2- and ASG7-dependent mechanisms.. Mol Genet Genomics.

[137] Hagen DC, McCaffrey G, Sprague GF (1991). Pheromone response elements are necessary and sufficient for basal and pheromone-induced transcription of the FUS1 gene of Saccharomyces cerevisiae.. Mol Cell Biol.

