# Supplementary figures and images for: Multiple Signals Converge on a Differentiation MAPK Pathway
Source: PLoS Genet. 2010 Mar 19;6(3):e1000883. doi: 10.1371/journal.pgen.1000883 (PMC2841618; doi:10.1371/journal.pgen.1000883)

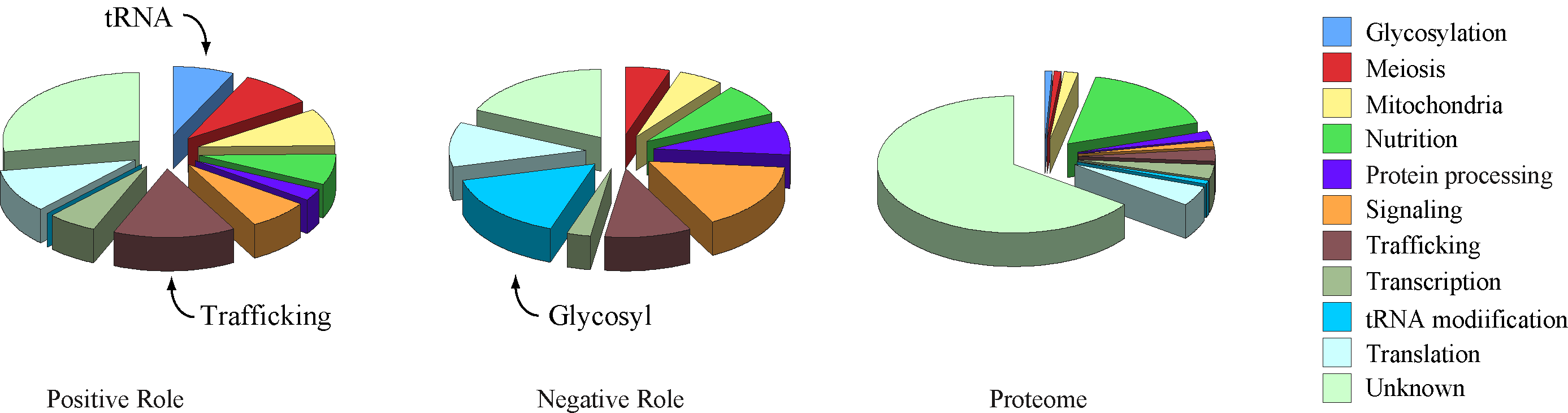

Supplement: Figure S1 — Genes that regulate Msb2p-HA secretion comprise a number of different functional categories. Pie charts of functional categories enriched in the genomic screens. Far left, functional categories that contributed to Msb2p-HA secretion; middle panel, functional categories that were inhibitory for Msb2p-HA secretion; far right, the overall functional classification of genes in the yeast genome for reference. Functional classification of yeast genes was facilitated by SGD (http://www.yeastgenome.org/). (6.08 MB TIF) [file pgen.1000883.s001.tif]

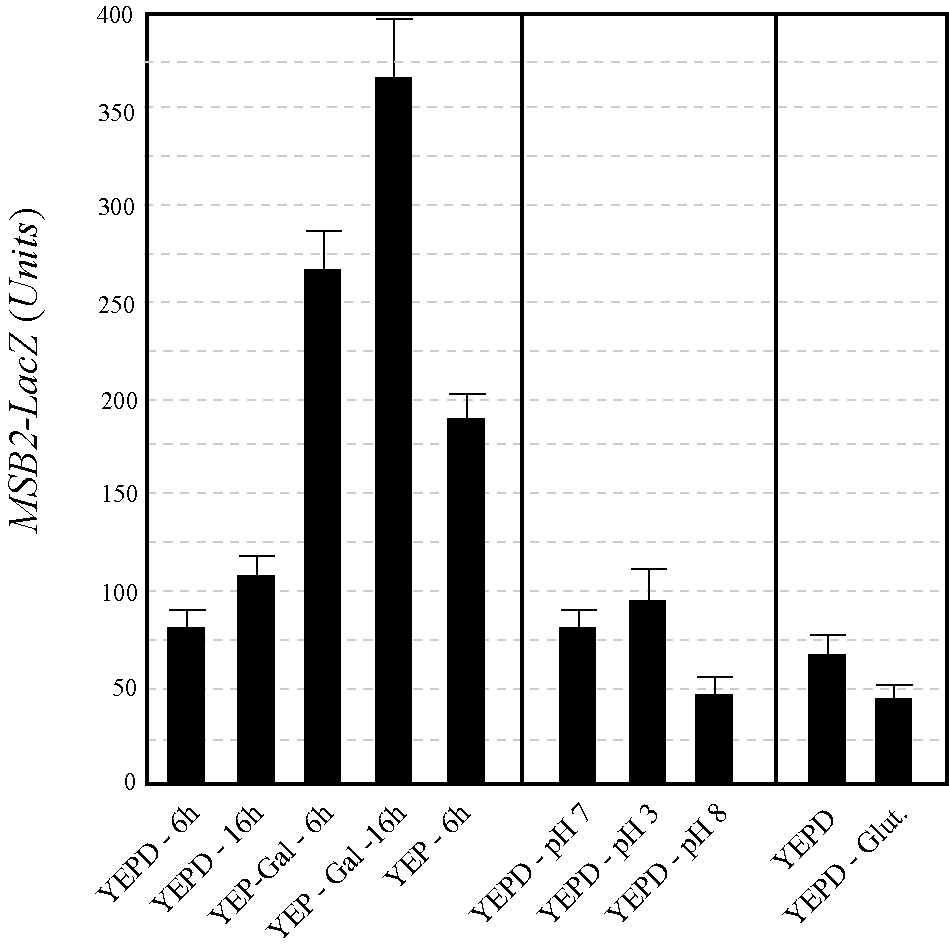

Supplement: Figure S2 — MSB2 expression is influenced by a different extracellular stimuli. The expression of an MSB2-lacZ fusion was examined under the conditions described. Cells were grown to mid-log phase in YEPD medium (∼6h) or medium lacking glucose (YEP), containing a poor carbon source (YEP-GAL), or to saturation in a poor carbon source (YEP-GAL 16h). MSB2-lacZ expression was also compared in medium supplemented with the amino acid glutamate and medium at pH 3, pH 7, and pH 8. Cells were harvested, and β-galactosidase assays were performed in independent replicates. The error bars represent standard deviation between experiments. (2.70 MB TIF) [file pgen.1000883.s002.tif]

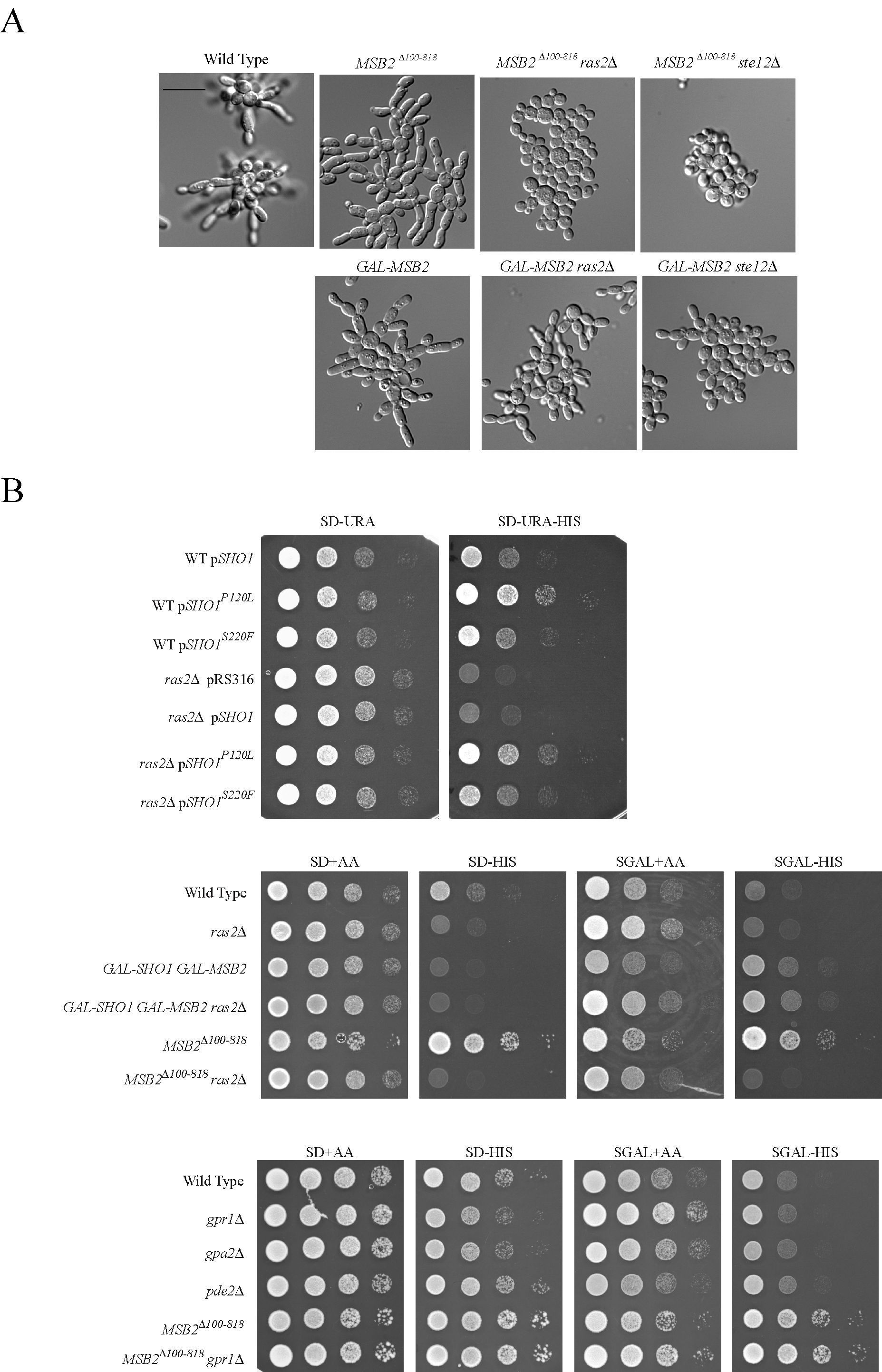

Supplement: Figure S3 — Genetic suppression analysis of RAS pathway components based on the activity of the FUS1-HIS3 reporter. (A) The morphology of wild-type cells and cells containing the activated allele MSB2Δ100–818 or overexpressing MSB2 (GAL-MSB2) in combination with ras2Δ and ste12Δ mutations. Images were taken by DIC at 100X. Bar, 20 microns. (B) Equal concentrations of cells of the indicated genotypes were spotted onto synthetic medium supplemented with glucose (SD) or galactose (S+GAL) that contained all amino acids (+AA), or that lacked uracil (-URA), and/or lacking histidine (-HIS). Plates were incubated at 30°C and spots were photographed. Growth on medium is indicative of the activity of the FG pathway. In the top panel, SHO1P120L and SHO1S22oF bypass the signaling defect of the ras2Δ mutant suggesting that Sho1p functions below Ras2p, and in line with the idea that Ras2p controls MSB2 expression. In the middle panel, overexpression of MSB2 bypasses the signaling defect of the ras2Δ mutant to a greater degree than the activated allele MSB2Δ100–818. In the bottom panel, Flo8p pathway mutants are not required to activate the FG pathway reporter. (3.55 MB TIF) [file pgen.1000883.s003.tif]

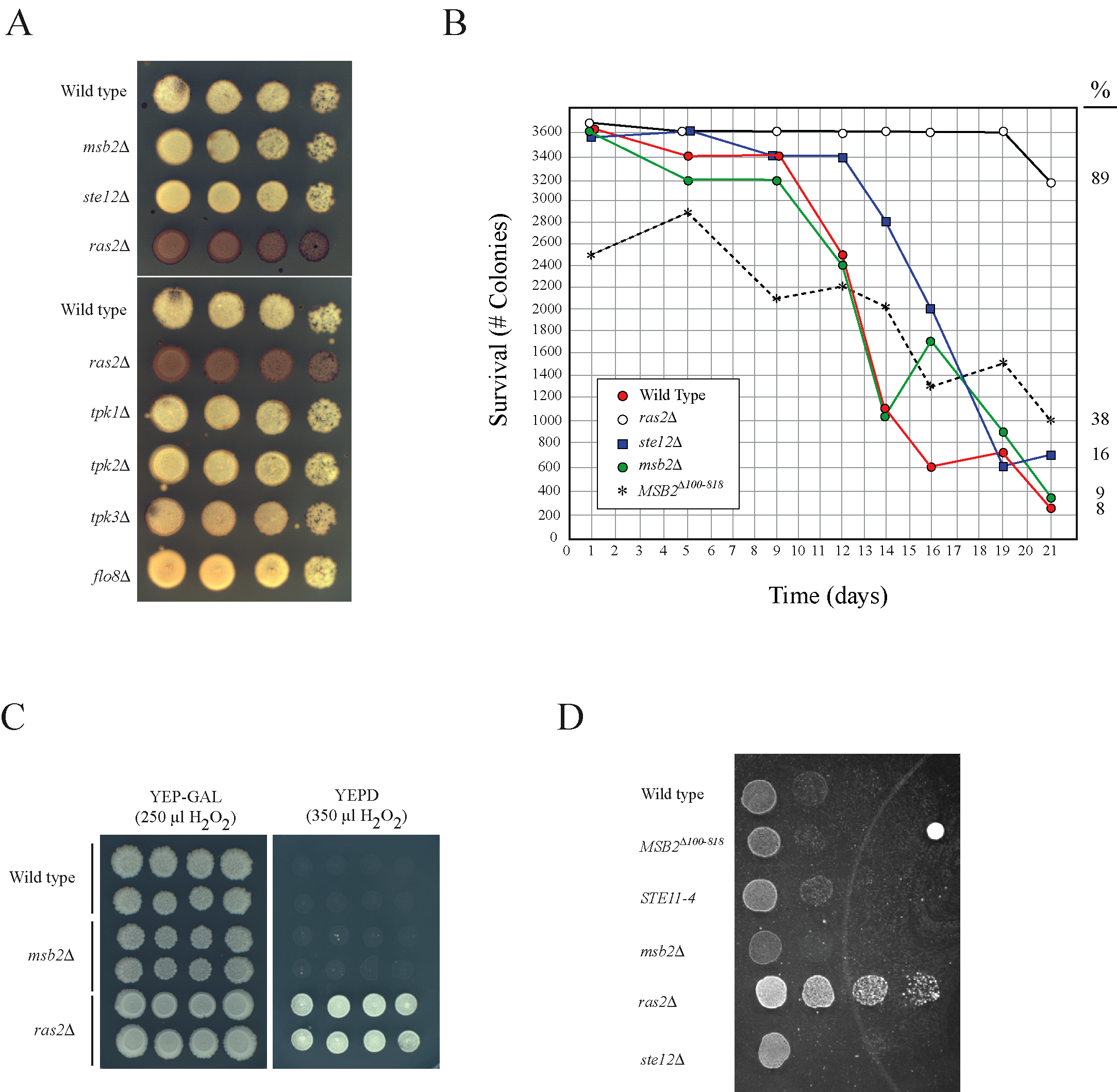

Supplement: Figure S4 — The FG pathway does not regulate RAS pathway outputs. (A) The ras2Δ mutant accumulates glycogen, whereas FG pathway mutants do not. Equal concentrations of cells of the indicated genotypes were spotted onto YEPD medium for 4d at 30°C. Plates were exposed to iodine vapor for ∼1 min and photographed. (B) Chronological survival of strains lacking Ras2p or FG MAPK components. Longevity was evaluated in YEPD medium over a 21-day time course. At the indicated days, a sample of the culture was removed and examined for the number of viable colonies by serial dilution on YEPD medium. (C) Sensitivity of RAS and MAPK components to oxidative stress. Equal concentrations of cells were spotted onto YEPD and YEP-GAL media containing the indicated volume of hydrogen peroxide. Plates were incubated for 2d at 30°C and photographed. (D) The ras2 mutant is resistant to oleate in comparison to wild-type cells and FG pathway mutants. Equal concentrations of cells were spotted onto media containing oleate and examined after 7d at 30°C. (2.72 MB TIF) [file pgen.1000883.s004.tif]

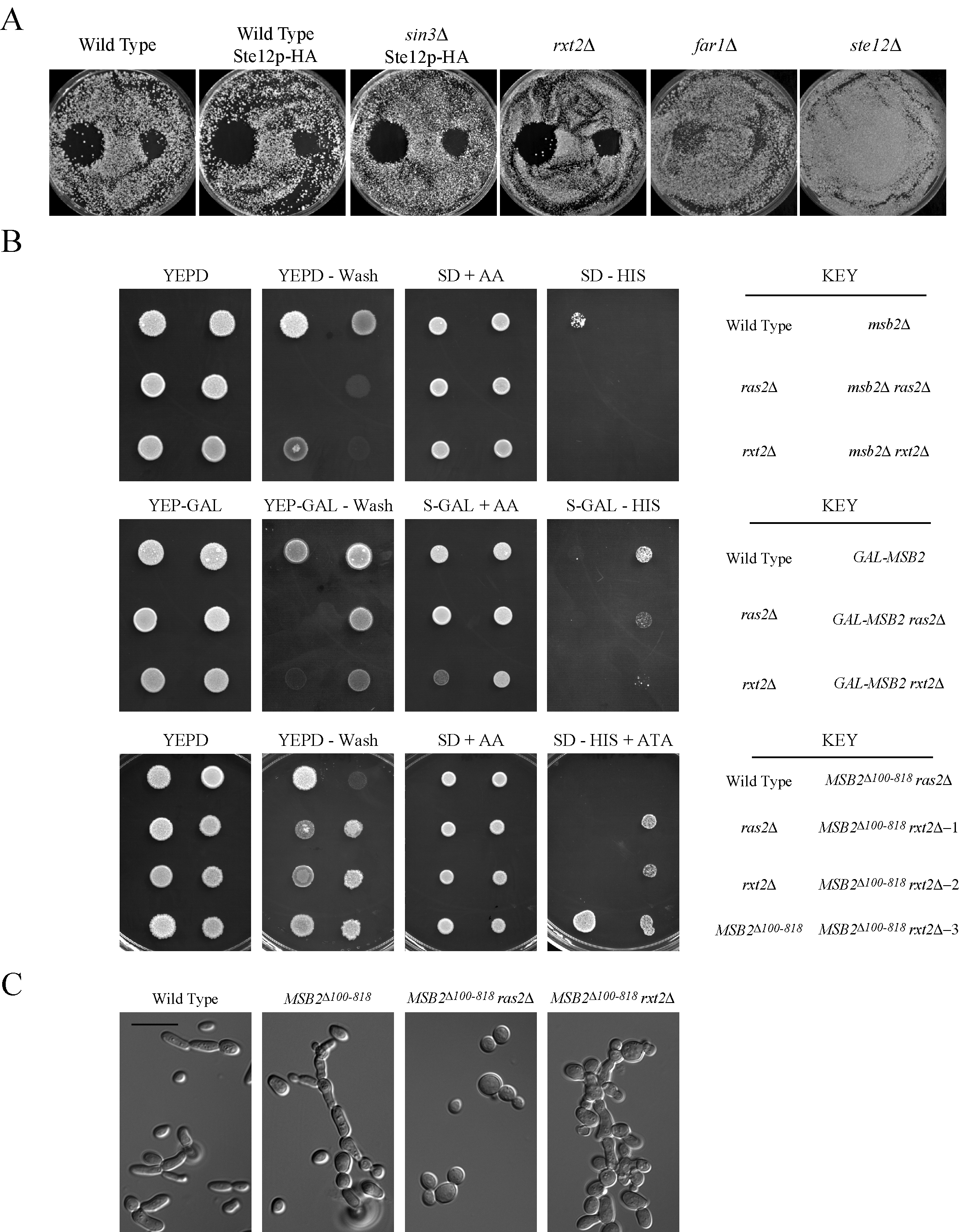

Supplement: Figure S5 — Genetic analysis of the Sin3p-Rpd3p complex in regulating MAPK signaling. (A) Halo assays. Wild-type cells and the indicated mutants were spread onto YEPD medium and 1 µl of 1uM α-factor was applied to plates. (B) Genetic suppression analysis of the rxt2Δ mutant in combination with other mutants in the filamentous growth pathway. (C) Cell morphologies of the ras2Δ and rxt2Δ mutants in combination with the activated allele MSB2Δ100–818. Bar, 10 microns. (4.03 MB TIF) [file pgen.1000883.s005.tif]

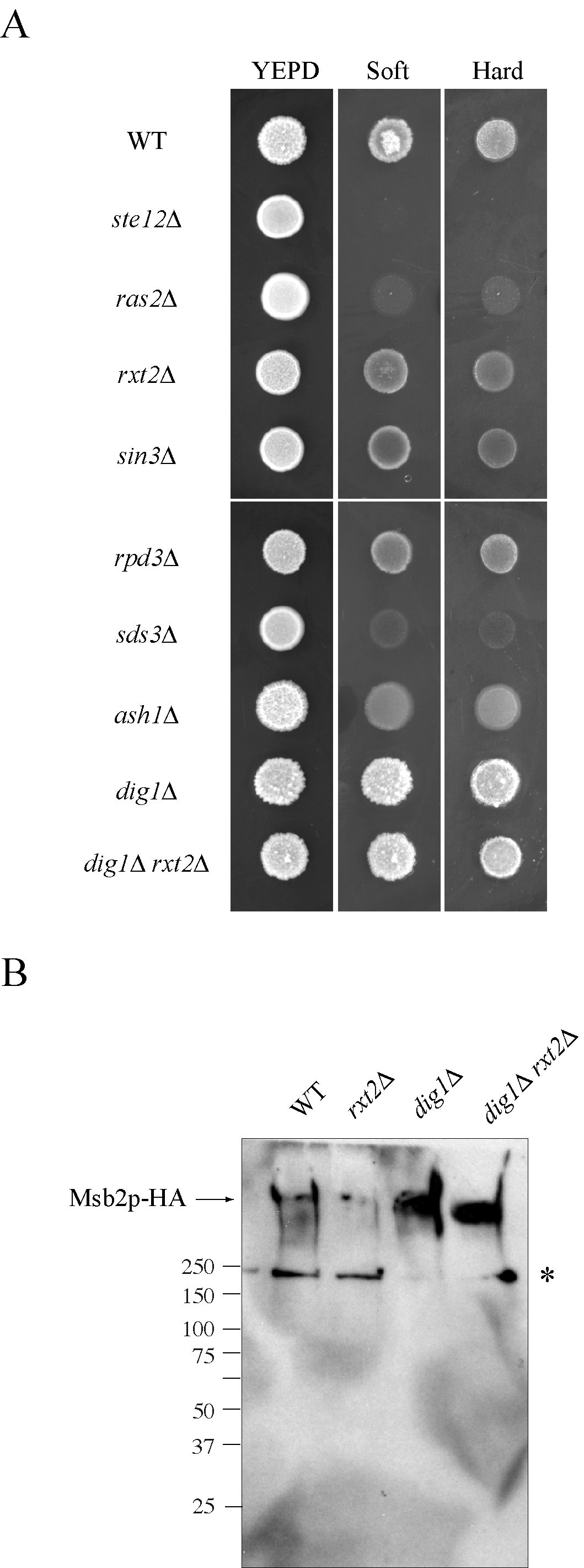

Supplement: Figure S6 — The Dig1p protein does not function through Rpd3(L). (A) Plate-washing assay showing the agar-invasion defects of Sin3p-Rpd3p complex mutants. (B) Immunoblot analysis of Msb2p-HA levels in wild-type cells and the dig1Δ rxt2Δ and dig1Δrxt2Δ double mutant. (9.03 MB TIF) [file pgen.1000883.s006.tif]

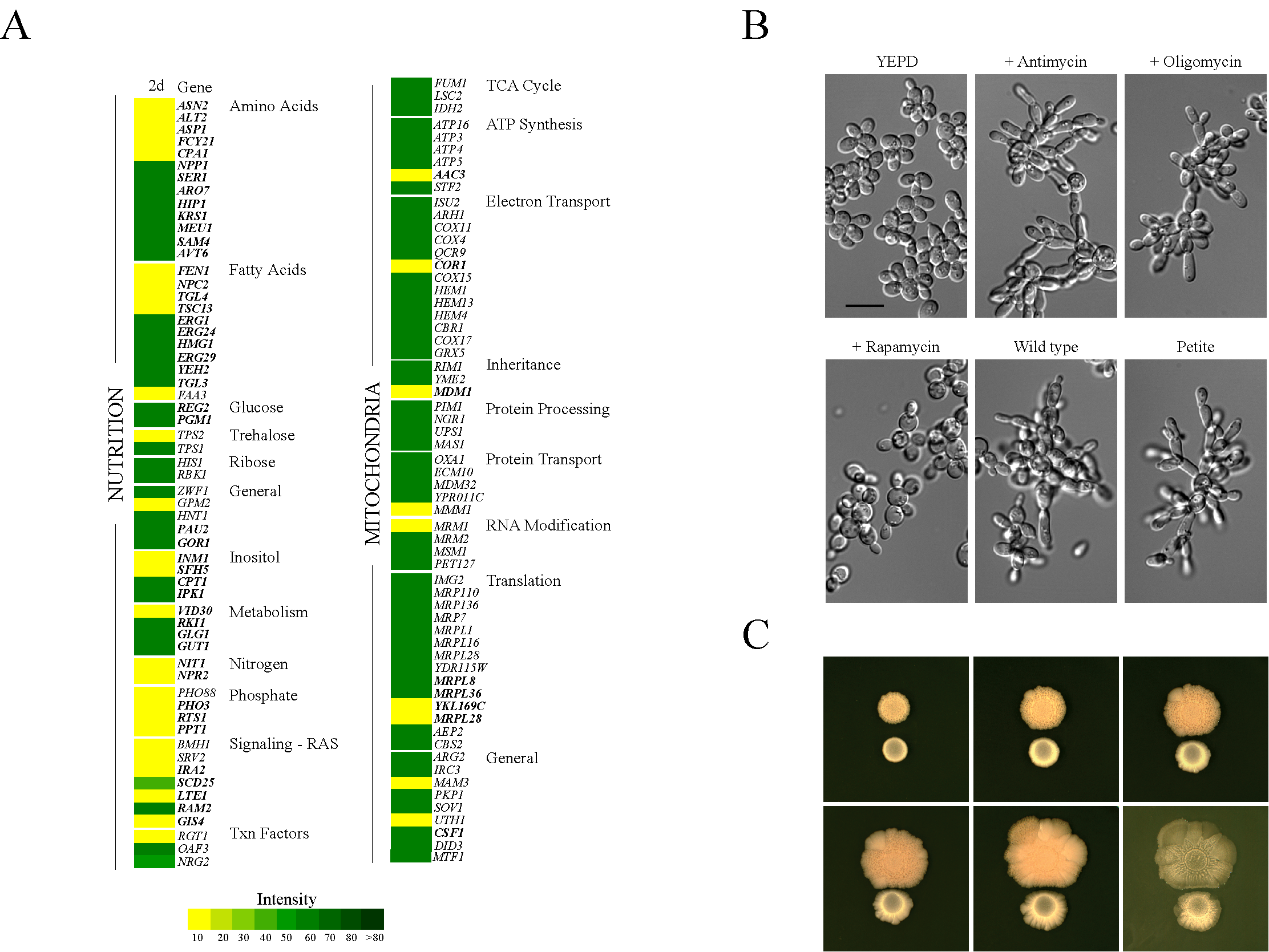

Supplement: Figure S7 — The relationship between filamentous growth regulation and cellular respiration. (A) Secretion profiling identifies nutritional regulatory and enzymatic genes and genes that function in respiration or mitochondrial functions. (B) The role of the mitochondria on filamentous growth. Antimycin and oligomycin, but not rapamycin induce filamentation. Petite mutants are capable of undergoing filamentous growth. Cells were grown on semi-solid agar medium for 24 h and assessed by microscopy for filamentous growth. Bar, 10 microns. (C) A functional mitochondria is required for mat expansion. Mats were incubated on YEPD medium and assessed over time for expansion. On the final day, the plate was washed to reveal invaded cells. (D) Expression of MSB2-lacZ in cells exposed to mitochondrial inhibitors antimycin or oligomycin or in peitie mutants. (11.14 MB TIF) [file pgen.1000883.s007.tif]

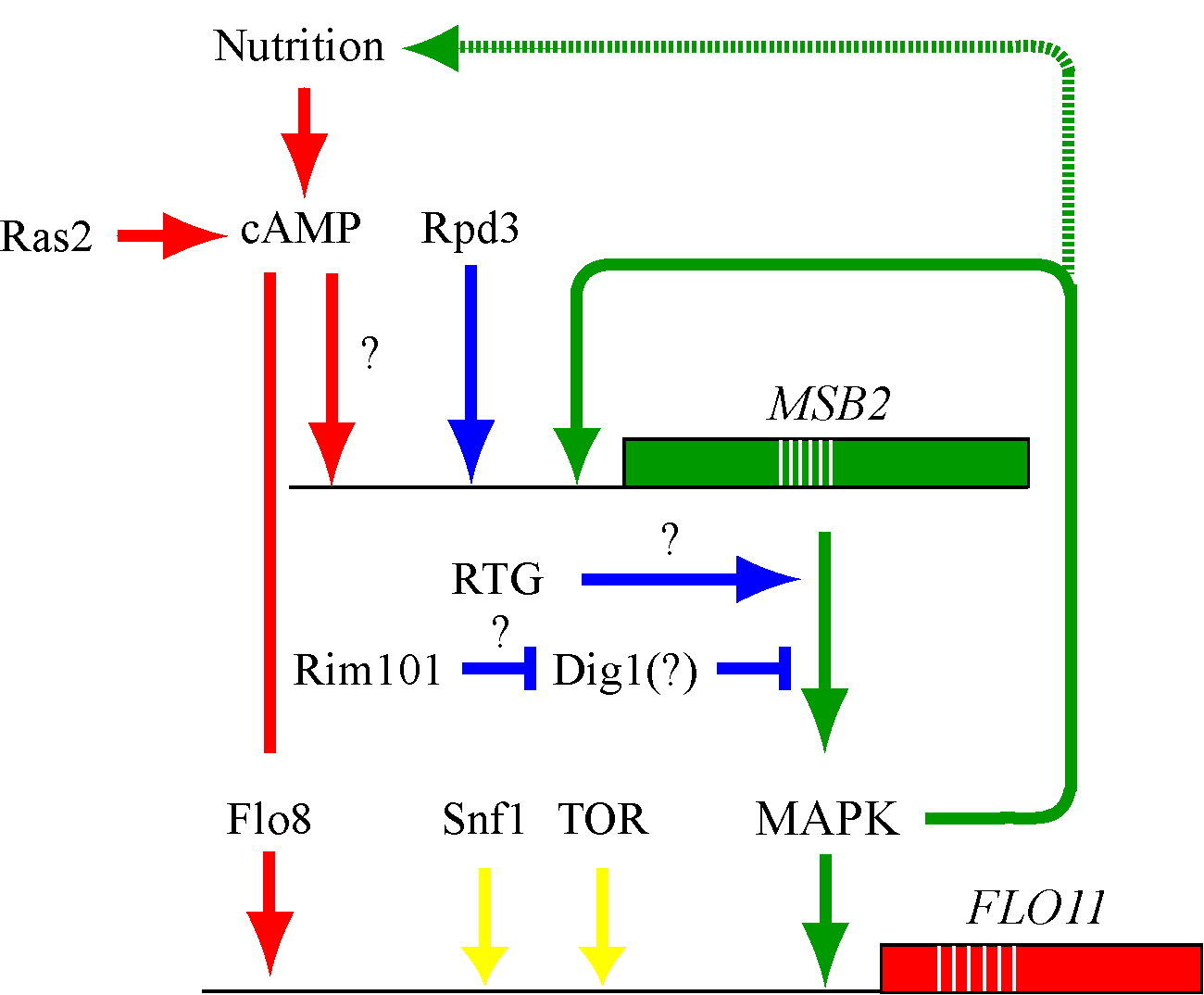

Supplement: Figure S8 — Model for the combinatorial regulation of the MSB2 and FLO11 promoters. Nutritional information is conveyed to the regulation of MSB2 expression through Ras2p/cAMP and Rpd3(L). Rpd3(L) is required MSB2 expression by association with the MSB2 promoter (blue line). Ras2p contributes to MAPK regulation by several mechanisms (red lines). Ras2p/cAMP is required to activate MSB2 expression through an unknown mechanism (denoted by question mark). In contrast, Ras2p-cAMP-PKA-Flo8p is required for FLO11 expression through the Flo8p transcription factor. Msb2p regulates FLO11 expression by MAPK signaling (green arrows). Msb2p further regulates its own expression through autofeedback by the MAPK pathway (green arrows). The dotted green line represents the feed-forward loop, by MAPK induction of genes that function in nutritional scavenging. FLO11-specific regulators are shown in yellow (Snf1p, TOR). (4.19 MB TIF) [file pgen.1000883.s008.tif]
