# Supplementary material for: Multiple Signals Converge on a Differentiation MAPK Pathway
Source: PLoS Genet. 2010 Mar 19;6(3):e1000883. doi: 10.1371/journal.pgen.1000883 (PMC2841618; doi:10.1371/journal.pgen.1000883)
Supplement: Table S1 — Yeast strains. (0.22 MB DOC) [file pgen.1000883.s009.doc]

Table S1. Yeast strains.

| Strain | Genotype | Source |
| --- | --- | --- |
| PC312 | *MATα ura3-52* | (Cullen and Sprague 2000) |
| PC313 | *MAT***a** *ura3-52* | (Cullen and Sprague 2000) |
| PC538^a^ | *MAT***a** *ste4 FUS1-lacZ FUS1-HIS3 ura3-52* | (Cull*en et a*l. 2004) |
| PC986^b^ | *MAT***a** *ura3-52 leu2 his3 trp1* | (Rober*ts et a*l. 2000) |
| BY4741^c^ | *his3*Δ0 *leu2*Δ0 *ura3*Δ0 *met15*Δ0 | Research Genetics |
| PC652 | *MATα ura3-52 leu2* | (Cullen and Sprague 2002) |
| PC999 | *MAT***a** *ste4 FUS1-lacZ FUS1-HIS3 ura3-52 MSB2-HA* | (Cull*en et a*l. 2004) |
| PC586 | *MATα ura3-52 leu2* | (Cull*en et a*l. 2004) |
| PC1531 | *MAT***a** *ste4 FUS1-lacZ FUS1-HIS3 ura3-52 sho1::HYG* | (Cull*en et a*l. 2004) |
| PC1079 | *MAT***a** *ste4 FUS1-lacZ FUS1-HIS3 ura3-52 GAL-MSB2 ste12::URA3* | (Cull*en et a*l. 2004) |
| PC948 | *MAT***a** *ste4 FUS1-lacZ FUS1-HIS3 ura3-52 msb2::KanMX6* | (Cull*en et a*l. 2004) |
| PC539 | *MAT***a** *ste4 FUS1-lacZ FUS1-HIS3 ura3-52 ste12::URA3* | (Pitoniak et al. 2009) |
| PC1029 | *MAT***a** *ste4 FUS1-lacZ FUS1-HIS3 ura3-52 flo11::KanMX6* | Karauranthi et al SUBMITTED |
| PC1083 | *MAT***a** *ste4 FUS1-lacZ FUS1-HIS3 ura3-52 GAL-MSB2-HA::KanMX6* | (Cull*en et a*l. 2004) |
| PC2043 | *MAT***a** *ste4 FUS1-lacZ FUS1-HIS3 ura3-52 FLO11-HA::KanMX6* | Karauranthi et al SUBMITTED |
| PC1516 | *MAT***a** *ste4 FUS1-lacZ FUS1-HIS3 ura3-52 MSB2^Δ100-818^* | (Vada*ie et a*l. 2008) |
| PC1658 | *MAT***a** *ura3-52 (wild-type control strain NY13)* | (TerBu*sh et a*l. 1996) |
| PC1661 | *MAT***a** *ura3-52 sec15-1 (NY64)* | (TerBu*sh et a*l. 1996) |
| PC1662 | *MAT***a** *ura3-52 sec5-24 (NY402)* | (TerBu*sh et a*l. 1996) |
| PC1523 | *MAT***a** *ste4 FUS1-lacZ FUS1-HIS3 ura3-52 ssk1::NAT* | (Pitoniak et al. 2009) |
| PC611 | *MAT***a** *ste4 FUS1-lacZ FUS1-HIS3 ura3-52 ste11::URA3* | (Pitoniak et al. 2009) |
| PC562 | *MAT***a** *ste4 FUS1-lacZ FUS1-HIS3 ura3-52 ras2::URA3* | This study |
| PC622 | *MAT***a** *ste4 FUS1-lacZ FUS1-HIS3 ura3-52 GAL-SHO1::KanMX6* | This study |
| PC673 | *MAT***a** *ste4 FUS1-lacZ FUS1-HIS3 ura3-52 ste20::KanMX6* | This study |
| PC687 | *MAT***a** *ste4 FUS1-lacZ FUS1-HIS3 sok2::KlURA3* | This study |
| PC1811 | *MAT***a** *ste4 FUS1-lacZ FUS1-HIS3 ura3-52 MSB2^Δ100-818^ ste12::URA3* | This study |
| PC1894 | *MAT***a** *ste4 FUS1-lacZ leu2::HYG FUS1-HIS3 ura3-52* | This study |
| PC2095 | *MAT***a** *ste4 FUS1-lacZ::NAT leu2::HYG FUS1-HIS3 ura3-52* | This study |
| PC2112 | *MAT***a** *ste4 FUS1-lacZ::NAT leu2::HYG FUS1-HIS3 ura3-52 tec1::LEU2* | This study |
| PC2130 | *MAT***a** *ste4 FUS1-lacZ::NAT leu2::HYG FUS1-HIS3 ura3-52 ste12::LEU2* | This study |
| PC2360 | *MAT***a** *ste4 FUS1-lacZ FUS1-HIS3 ura3-52 ras2::NAT* | This study |
| PC2361 | *MAT***a** *ste4 FUS1-lacZ FUS1-HIS3 ura3-52 leu2::HYG ras2::NAT* | This study |
| PC2362 | *MAT***a** *ste4 FUS1-lacZ FUS1-HIS3 ura3-52 ira1::NAT* | This study |
| PC2363 | *MAT***a** *ste4 FUS1-lacZ FUS1-HIS3 ura3-52 gpa2::NAT* | This study |
| PC2364 | *MAT***a** *ste4 FUS1-lacZ FUS1-HIS3 ura3-52 MSB2-HA^Δ100-818^ ras2::URA3* | This study |
| PC2366 | *MAT***a** *ste4 FUS1-lacZ FUS1-HIS3 ura3-52 MSB2-HA^Δ100-818^ gpa2::NAT (unconfirmed)* | This study |
| PC2367 | *MAT***a** *ste4 FUS1-lacZ FUS1-HIS3 ura3-52 MSB2-HA^Δ100-818^ tpk2::NAT (unconfirmed)* | This study |
| PC2368 | *MAT***a** *ste4 FUS1-lacZ FUS1-HIS3 ura3-52 GAL-SHO1::KanMX6 gpa2::NAT (unconfirmed)* | This study |
| PC2369 | *MAT***a** *ste4 FUS1-lacZ FUS1-HIS3 ura3-52 GAL-SHO1::KanMX6 ira1::NAT (unconfirmed)* | This study |
| PC2510 | *MAT***a** *ste4 FUS1-lacZ FUS1-HIS3 ura3-52 ira1::NAT ste12::URA3* | This study |
| PC2511 | *MAT***a** *ste4 FUS1-lacZ FUS1-HIS3 ura3-52 ras2::NAT ste12::URA3* | This study |
| PC2513 | *MATa leu2::ura3 ras2::HYG* | This study |
| PC2515 | *MATa leu2::ura3 flo8::HYG* | This study |
| PC2516 | *MATa leu2::ura3 ira1::HYG* | This study |
| PC2519 | *MAT***a** *ste4 fus1-lacZ::NAT FUS1-HIS3 ura3-52* | This study |
| PC2520 | *MAT***a** *ste4 fus1-lacZ::NAT FUS1-HIS3 ura3-52 msb2::URA3* | This study |
| PC2522 | *MAT***a** *ste4 fus1-lacZ::NAT FUS1-HIS3 ura3-52 sho1::HYG* | This study |
| PC2523 | *MAT***a** *ste4 fus1-lacZ::NAT FUS1-HIS3 ura3-52 flo8::HYG* | This study |
| PC2524 | *MAT***a** *ste4 FUS1-lacZ FUS1-HIS3 ura3-52 ira1::NAT ras2::URA3* | This study |
| PC2526 | *MAT***a** *ste4 FUS1-lacZ FUS1-HIS3 ura3-52 GAL-SHO1::KanMX6 ira1::NAT ste12::URA3 (confirmed?)* | This study |
| PC2527 | *MAT***a** *ste4 FUS1-lacZ FUS1-HIS3 ura3-52 ira1::NAT TUB1-GFP::URA3* | This study |
| PC2532 | *MAT***a** *ste4 FUS1-lacZ FUS1-HIS3 ura3-52 flo8::HYG* | This study |
| PC2534 | *MAT***a** *ste4 FUS1-lacZ FUS1-HIS3 ura3-52 pde2::HYG* | This study |
| PC2535 | *MAT***a** *ste4 FUS1-lacZ FUS1-HIS3 ura3-52 gpa2::NAT* | This study |
| PC2536 | *MAT***a** *ste4 FUS1-lacZ FUS1-HIS3 ura3-52 MSB2-HA^Δ100-818^ gpr1::URA3* | This study |
| PC2537 | *MAT***a** *ste4 FUS1-lacZ FUS1-HIS3 ura3-52 gpr1:: KlURA3* | This study |
| PC2538 | *MAT***a** *ste4 FUS1-lacZ FUS1-HIS3 ura3-52 leu2::HYG STE11-4* | This study |
| PC2539 | *MAT***a** *ste4 FUS1-lacZ FUS1-HIS3 ura3-52 ras2::NAT STE11-4* | This study |
| PC2540 | *MAT***a** *ste4 FUS1-lacZ FUS1-HIS3 ura3-52 sho1::HYG STE11-4* | This study |
| PC2541 | *MAT***a** *ste4 FUS1-HIS3 ura3-52 STE20^ΔPAK^ STE11-4* | This study |
| PC2542 | *MAT***a** *ste4 FUS1-lacZ FUS1-HIS3 ura3-52 ira1::NAT ste11::URA3* | This study |
| PC2543 | *MAT***a** *ste4 FUS1-lacZ FUS1-HIS3 ura3-52 ras2::NAT ste11::URA3* | This study |
| PC2544 | *MAT***a** *ste4 FUS1-lacZ FUS1-HIS3 ura3-52 ste11::URA3* | This study |
| PC2545 | *MAT***a** *ste4 fus1-lacZ::NAT FUS1-HIS3 ura3-52 ste12::KanMX6* | This study |
| PC2546 | *MAT***a** *ste4 fus1-lacZ::NAT FUS1-HIS3 ura3-52 MSB2-HA^Δ100-818^* | This study |
| PC2549 | *MAT***a**  *ura3-52 ras2::URA3* | This study |
| PC2588 | *MAT***a** *ste4 FUS1-lacZ FUS1-HIS3 ura3-52 tpk1::NAT* | This study |
| PC2589 | *MATα leu2::ura3 tpk1::NAT* | This study |
| PC2590 | *MAT***a** *ste4 FUS1-lacZ FUS1-HIS3 ura3-52 tpk2::NAT* | This study |
| PC2591 | *MATα leu2::ura3 tpk2::NAT* | This study |
| PC2592 | *MAT***a** *ste4 FUS1-lacZ FUS1-HIS3 ura3-52 tpk3::NAT* | This study |
| PC2593 | *MATα leu2::ura3 tpk3::NAT* | This study |
| PC2633 | *MAT***a** *ste4 FUS1-lacZ FUS1-HIS3 ura3-52 sdc25::NAT* | This study |
| PC2634 | *MAT***a** *ste4 FUS1-lacZ FUS1-HIS3 ura3-52 azf1::NAT* | This study |
| PC2635 | *MATα leu2::ura3 sdc25::NAT* | This study |
| PC2636 | *MATα leu2::ura3 azf1::NAT* | This study |
| PC2687 | *MAT***a** *ste4 FUS1-lacZ FUS1-HIS3 ura3-52 MSB2-HA ste12::URA3* | This study |
| PC2689 | *MAT***a** *ste4 FUS1-lacZ FUS1-HIS3 ura3-52 MSB2-HA ras2::URA3* | This study |
| PC2691 | *MAT***a** *ste4 FUS1-lacZ FUS1-HIS3 ura3-52 FLO11-HA ste12::URA3* | This study |
| PC2693 | *MAT***a** *ste4 FUS1-lacZ FUS1-HIS3 ura3-52 FLO11-HA ras2::URA3* | This study |
| PC2840 | *MAT***a** *ste4 FUS1-lacZ FUS1-HIS3 ura3-52* *FLO11-HA rxt2:: KlURA3* | This study |
| PC2841 | *MAT***a** *ste4 FUS1-lacZ FUS1-HIS3 ura3-52* *FLO11-HA rim101:: KlURA3* | This study |
| PC2842 | *MAT***a** *ste4 FUS1-lacZ FUS1-HIS3 ura3-52* *FLO11-HA hda1:: KlURA3* | This study |
| PC2843 | *MAT***a** *ste4 FUS1-lacZ FUS1-HIS3 ura3-52* *FLO11-HA mss11:: KlURA3* | This study |
| PC2844 | *MAT***a** *ste4 FUS1-lacZ FUS1-HIS3 ura3-52* *FLO11-HA snf2:: KlURA3* | This study |
| PC2845 | *MAT***a** *ste4 FUS1-lacZ FUS1-HIS3 ura3-52* *FLO11-HA gal11:: KlURA3* | This study |
| PC2846 | *MAT***a** *ste4 FUS1-lacZ FUS1-HIS3 ura3-52* *FLO11-HA msn1:: KlURA3* | This study |
| PC2847 | *MAT***a** *ste4 FUS1-lacZ FUS1-HIS3 ura3-52* *FLO11-HA opi1:: KlURA3* | This study |
| PC2849 | *MAT***a** *ste4 FUS1-lacZ FUS1-HIS3 ura3-52* *FLO11-HA yak1:: KlURA3* | This study |
| PC2927 | *MAT***a** *ste4 fus1-lacZ FUS1-HIS3 MSB2-HA ras2::NAT ura3-52* | This study |
| PC2945 | *MAT***a** *ste4 FUS1-lacZ FUS1-HIS3 ura3-52* *MSB2-HA rxt2::NAT* | This study |
| PC2946 | *MAT***a** *ste4 FUS1-lacZ FUS1-HIS3 ura3-52* *GAL-MSB2::KANMX6 rxt2:: NAT* | This study |
| PC2947 | *MAT***a** *ste4 FUS1-lacZ FUS1-HIS3 ura3-52 MSB2-HA ^Δ100-818^* *rxt2::NAT* | This study |
| PC2948 | *MAT*α *leu2::ura3 rxt2::NAT* | This study |
| PC2949 | *MAT***a** *ste4 FUS1-lacZ FUS1-HIS3 ura3-52 GAL-MSB2::KanMX6 ras2::URA3* | This study |
| PC2950 | *MAT***a** *ura3-53 rxt2::NAT* | This study |
| PC2951 | *MAT***a** *ste4 FUS1-lacZ FUS1-HIS3 ura3-52* *MSB2-HA gal11:: KlURA3* | This study |
| PC2952 | *MAT***a** *ste4 FUS1-lacZ FUS1-HIS3 ura3-52* *MSB2-HA hda1:: KlURA3* | This study |
| PC2953 | *MAT***a** *ste4 FUS1-lacZ FUS1-HIS3 ura3-52* *MSB2-HA rim101:: KlURA3* | This study |
| PC2954 | *MAT***a** *ste4 FUS1-lacZ FUS1-HIS3 ura3-52* *MSB2-HA snf2:: KlURA3* | This study |
| PC2955 | *MAT***a** *ste4 FUS1-lacZ FUS1-HIS3 ura3-52* *MSB2-HA msn1:: KlURA3* | This study |
| PC2956 | *MAT***a** *ste4 FUS1-lacZ FUS1-HIS3 ura3-52* *MSB2-HA yak1:: KlURA3* | This study |
| PC2957 | *MAT***a** *ste4 FUS1-lacZ FUS1-HIS3 ura3-52* *MSB2-HA mss11:: KlURA3* | This study |
| PC3021 | *MAT***a** *STE12-HA::KanMX6* | This study |
| PC3030 | *MAT***a** *ste4 FUS1-lacZ FUS1-HIS3 ura3-52* *MSB2-HA sin3:: NAT* | This study |
| PC3031 | *MAT***a** *ste4 FUS1-lacZ FUS1-HIS3 ura3-52* *MSB2-HA isw1::NAT* | This study |
| PC3032 | *MAT***a** *ste4 FUS1-lacZ FUS1-HIS3 ura3-52* *MSB2-HA cka1::NAT* | This study |
| PC3033 | *MAT***a** *ste4 FUS1-lacZ FUS1-HIS3 ura3-52* *MSB2-HA nhp10::NAT* | This study |
| PC3034 | *MAT***a** *ste4 FUS1-lacZ FUS1-HIS3 ura3-52* *MSB2-HA isw2::NAT* | This study |
| PC3035 | *MAT***a** *ste4 FUS1-lacZ FUS1-HIS3 ura3-52* *MSB2-HA mks1::NAT* | This study |
| PC3036 | *MAT***a** *ste4 FUS1-lacZ FUS1-HIS3 ura3-52* *MSB2-HA ash1:: KlURA3* | This study |
| PC3037 | *MAT***a** *ste4 FUS1-lacZ FUS1-HIS3 ura3-52* *MSB2-HA sds3:: KlURA3* | This study |
| PC3038 | *MAT***a** *ste4 FUS1-lacZ FUS1-HIS3 ura3-52* *MSB2-HA rpd3:: KlURA3* | This study |
| PC3039 | *MAT***a** *ste4 FUS1-lacZ FUS1-HIS3 ura3-52* *MSB2-HA dig1:: KlURA3* | This study |
| PC3040 | *MAT***a** *ste4 FUS1-lacZ FUS1-HIS3 ura3-52* *MSB2-HA rxt2::NAT dig1:: KlURA3* | This study |
| PC3095 | *MATα leu2::ura3 TRX3-CFP::KanMX6 URA3* | This study |
| PC3096 | *MATα leu2::ura3 pde2::NAT* | This study |
| PC3347 | *MAT***a** *ste4 FUS1-lacZ FUS1-HIS3 DIG1-HA::KanMX6* | This study |
| PC3349 | *MAT***a** *ste4 FUS1-lacZ FUS1-HIS3 DIG1-HA::KanMX6 rxt2::KlURA3* | This study |
| PC3351 | *MAT***a** *STE12-HA::KanMX6 rxt2::KlURA3* | This study |
| PC3352 | *MAT***a** *STE12-HA::KanMX6 ras2::NAT* | This study |
| PC3353 | *MAT***a** *STE12-HA::KanMX6 sin3::NAT* | This study |
| PC3358 | *MAT***a** *ste4 FUS1-lacZ FUS1-HIS3 cla4::KlURA3* | This study |
| PC3359 | *MAT*α *leu2::ura3 mks1::NAT* | This study |
| PC3360 | *MAT*α *leu2::ura3 tpk1::NAT tpk2::KlURA3* | This study |
| PC3361 | *MAT*α *leu2::ura3 tpk2::KlURA3 tpk3::NAT* | This study |
| PC3362 | *MAT***a** *ste4 FUS1-lacZ FUS1-HIS3 MSB2-HA fkh1::KlURA3* | This study |
| PC3363 | *MAT***a** *ste4 FUS1-lacZ FUS1-HIS3 MSB2-HA nrg1::KlURA3* | This study |
| PC3427 | *MAT***a** *ste4 FUS1-lacZ FUS1-HIS3 MSB2-HA pde2::NAT* | This study |
| PC3428 | *MAT***a** *ste4 FUS1-lacZ FUS1-HIS3 MSB2-HA swi4::KlURA3* | This study |
| PC3429 | *MAT***a** *ste4 FUS1-lacZ FUS1-HIS3 MSB2-HA mga1::KlURA3* | This study |
| PC3430 | *MAT***a** *ste4 FUS1-lacZ FUS1-HIS3 MSB2-HA fkh2::NAT* | This study |
| PC3431 | *MAT***a** *ste4 FUS1-lacZ FUS1-HIS3 MSB2-HA sfl1::URA3* | This study |
| PC3432 | *MAT***a** *ste4 FUS1-lacZ FUS1-HIS3 MSB2-HA fkh1::URA3 fkh2::NAT* | This study |
| PC3433 | *MAT***a** *ste4 FUS1-lacZ FUS1-HIS3 MSB2-HA pde2::NAT ras2::URA3* | This study |
| PC3435 | *MAT***a** *ste4 FUS1-lacZ FUS1-HIS3 MSB2-HA nrg1::KlURA3 nrg2::NAT* | This study |
| PC3579 | *MAT***a** *SIN3-HA* | This study |
| PC3642 | *MAT***a** *ste4 FUS1-lacZ FUS1-HIS3 MSB2-HA rtg3::NAT* | This study |
| PC3643 | *MAT***a** *ste4 FUS1-lacZ FUS1-HIS3 MSB2-HA tco89::NAT* | This study |
| PC3644 | *MAT***a** *ste4 FUS1-lacZ FUS1-HIS3 MSB2-HA gzf3::NAT* | This study |
| PC3652 | *MAT***a** *ste4 FUS1-lacZ FUS1-HIS3 MSB2-HA rtg2::NAT* | This study |
| PC3654 | *MAT***a** *ste4 FUS1-lacZ FUS1-HIS3 MSB2-HA tor1::NAT* | This study |

a. All strains are in the Σ1278b background unless otherwise indicated.

b. S288c background ordered deletion collection control strain.

c. Deletion strains from the *MAT***a** ordered deletion collection in the S288c background were also used in this study.
