# Supplementary material for: Multiple Signals Converge on a Differentiation MAPK Pathway
Source: PLoS Genet. 2010 Mar 19;6(3):e1000883. doi: 10.1371/journal.pgen.1000883 (PMC2841618; doi:10.1371/journal.pgen.1000883)
Supplement: Table S2 — Plasmids used in this study. (0.08 MB DOC) [file pgen.1000883.s010.doc]

Table S2. Plasmids used in this study.

| Plasmid | Description | Source |
| --- | --- | --- |
| pRS316 | *CEN/URA3* parent plasmid | (Sikorski and Hieter 1989) |
| pRS315 | *CEN/LEU2* parent plasmid | (Sikorski and Hieter 1989) |
| pRS305 | *LEU2* parent plasmid for integration | (Sikorski and Hieter 1989) |
| p*FLO8* | YEplac181-FLO8 URA3 | (L*iu et a*l. 1996) |
| *pYLR042c-lacZ* | *FG* pathway reporter | (Rober*ts et a*l. 2000) |
| *pSVS1-lacZ* | *FG* pathway reporter | (Rober*ts et a*l. 2000) |
| *pKSS1-lacZ* | *FG* pathway reporter | (Rober*ts et a*l. 2000) |
| *pTy[FG]-lacZ* | *FG* pathway reporter | (Lalo*ux et a*l. 1994) |
| *pPGU1-lacZ* | *FG* pathway reporter | (Rober*ts et a*l. 2000) |
| *pFRE-lacZ* | *FG* pathway reporter | (Madhani and Fink 1997) |
| *pRS305 lacZ* | plasmid for *lacZ* gene integration (PC3364) | This Study |
| *pMSB2-lacZ* | V84 CEN/URA3-based plasmid | (Pitoni*ak et a*l. 2009) |
| *pMSB2-HA* | Internal tag at position 500 aa residues | (Vada*ie et a*l. 2008) |
| *pMSB2-GFP* | C-terminal fusion | (Vada*ie et a*l. 2008) |
| *pMSB2^AG^-lacZ* | V84 CEN/URA3-point mutation in the *STE12* binding site | (Pitoni*ak et a*l. 2009) |
|  |  |  |
